# Genetic characterization of *Listeria monocytogenes* strains from the Seeliger’s historical “special Listeria culture collection” (1918–1986)

**DOI:** 10.3389/fmicb.2026.1909666

**Published:** 2026-09-16

**Authors:** Beatriz Daza Prieto, Ariane Pietzka, Johannes Benjamin Stielow, Anna Lennkh, Andrea Murer, David Eisele, Tobias Mösenbacher, Anna Stöger, Patrick Hyden, Sven Halbedel, Franz Allerberger, Herbert Hof, Werner Ruppitsch

**Affiliations:** 1 Institute of Medical Microbiology and Hygiene, Austrian Agency for Health and Food Safety, Vienna, Austria; 2 National Reference Laboratory for Listeria, Division for Public Health, Austrian Agency for Health and Food Safety, Graz, Austria; 3 Institute of Hygiene and Medical Microbiology, Medical University of Innsbruck, Innsbruck, Austria; 4 Institute for Medical Microbiology and Hospital Hygiene, Robert Koch Institute, Wernigerode, Germany; 5 Institute of Molecular and Clinical Immunology, Otto-von-Guericke University Magdeburg, Magdeburg, Germany; 6 MVZ Labor Limbach and Colleagues, Heidelberg, Germany; 7 Faculty of Food Technology, Food Safety, and Ecology, University of Donja Gorica, Podgorica, Montenegro

**Keywords:** antimicrobial resistance genes, *Listeria monocytogenes*, orthogroups, virulence genes, whole genome sequencing

## Abstract

**Introduction:**

*Listeria monocytogenes* (*L. monocytogenes*) is a widespread foodborne pathogen that continues to pose a major challenge to public health and the food industry.

**Methods:**

To explore the genomic diversity of historical isolates, we used whole genome sequencing (WGS) both short- and long-read technologies to obtain high quality genomes of 64 historical *L. monocytogenes* isolates collected between 1918 and 1986. These isolates originated from environmental, animal, and human sources obtained from the collection from Professor Seeliger.

**Results:**

The isolates displayed a substantial genetic diversity, representing 15 clonal complexes (CCs), 40 sequence types (STs) and 59 core genome multilocus sequence types (cgMLST). The most frequently identified STs were ST2 (*n* = 8), ST7 (*n* = 6) and ST9 (*n* = 4). All isolates harbored known virulence genes, while 35 isolates contained mobile genetic elements (MGEs), ranging from 1 to 11 per isolate. Ten isolates also carried a plasmid. Every isolate possessed the intrinsic resistance genes *fosX* and *lin*, and none carried acquired antimicrobial resistance genes (ARGs). Accessory genome diversity mainly followed phylogenetic positioning, and rendered limited within, but not between lineage variability.

**Discussion/conclusion:**

WGS revealed high genetic diversity among the Seeliger *L. monocytogenes* isolates. Their close relatedness to contemporary isolates underscores the strong adaptability across sources and time, emphasizing the need for better prevention strategies.

## 1 Introduction

*L. monocytogenes* is the bacterium responsible for listeriosis in both humans and animals. In humans, this disease typically manifests in three severe invasive forms: bloodstream infection, involvement of the central nervous system, and maternal-fetal listeriosis (Hernandez-Milian and Payeras-Cifre, 2014). *L. monocytogenes* is strongly associated with foodborne transmission and is frequently isolated from ready-to-eat products, meat, milk, fish and seafood, ice cream, as well as fresh, frozen or processed vegetables, fruits and spices. However, this pathogen is widely distributed in nature, thriving in environments such as soil, water, vegetation, animal feed, agricultural settings and food-processing facilities (Daza Prieto et al., 2024). Once established, the bacterium can disseminate through aerosols, personnel, food-handling activities and contaminated contact surfaces, factors that contribute to its persistence and reduced susceptibility to routine cleaning and disinfection. Additionally, *L. monocytogenes* can form robust biofilms, a feature that enhances its long-term survival and promotes repeated cross-contamination of food products (Daza Prieto et al., 2024).

The extensive misuse of antibiotics in human and veterinary medicine has contributed to a rise in multidrug-resistant *L. monocytogenes* strains over recent decades (Maung et al., 2023). Although *L. monocytogenes* is generally susceptible to a broad range of antimicrobial agents, multidrug-resistant strains have been detected in food and environmental samples (Maung et al., 2023), following the first report of such strains in France in 1988 (Poyart-Salmeron et al., 1990). Recent studies indicate that the most frequently reported antibiotic resistances in *L. monocytogenes* include resistance against tetracycline, clindamycin and oxacillin (Tayeb Bizhar et al., 2023). In contrast, other research reported that the proportion of multidrug resistant strains identified in 2022 was significantly lower than that detected a decade earlier (Maung et al., 2023), suggesting temporal or geographical differences in antimicrobial resistance (AMR) patterns and highlighting the importance of continuous antimicrobial resistance surveillance. Additionally, virulence factors encoded by virulence genes (VGs) play a central role in the ability of *L. monocytogenes* to adapt, persist and spread in the environment. Listeria Pathogenicity Islands (LIPIs) are genomic regions that contribute significantly to virulence, with four LIPIs currently described, each containing genes involved in different stages of infections.

The Seeliger special collection represents an exceptional source for studying natural evolution, as it brings together *L. monocytogenes* isolates collected over several decades and sources, offering a continuous and unique view of their genetic changes over time. Between 1918 and 1986, Professor H. P. Seeliger (University of Würzburg, Germany) gathered *L. monocytogenes* isolates from a variety of sources—including food, humans, animals and the environment—thus creating the so-called “Special Listeria Culture Collection” (SLCC). This archive is an extraordinary resource for evolution-oriented studies (Haase et al., 2011). Sixty-four of the oldest isolates were successfully revived and subjected to whole-genome sequencing, generating high-resolution data that enable in-depth analyses of genetic diversity, evolutionary relationships, historical outbreaks and long-term trends in *L. monocytogenes* adaptation and AMR.

## 2 Materials and methods

### 2.1 Origin and sample collection

The Seeliger “Special Listeria Culture Collection” (SLCC), comprising more than 6,000 isolates, was collected by Professor H. P. Seeliger at the University of Würzburg, Germany and contains isolates from 1918 until the 1980s. Over several decades, Seeliger gathered thousands of *Listeria* isolates from across Germany and from numerous collaborators before his retirement in 1989. In 1991, the entire collection was transferred to Professor Herbert Hof at the University of Heidelberg, Germany, which was later preserved and rescued by Professor Mark Achtman at the University College Cork, Ireland (Haase et al., 2011). The strain information was recorded manually either handwritten or typed primarily in German. In 2013, the SLCC (stab cultures and lyophilized material) was transferred from Cork to the National Reference Laboratory for Listeria (NRL) at the Austrian Agency for Health and Food Safety in Graz, Austria.

### 2.2 Cultural detection of *L. monocytogenes*

#### 2.2.1 Resuscitation of cultures

*Lyophils*: tubes were opened with a glass file and tryptose-phosphate-bouillon was added using a pipette. The material was aspirated, transferred to a tryptose-phosphate-bouillon tube, incubated over night at 37 °C and on the next day spread on Columbia agar with 5% sheep blood (COS) plates (Biomerieux, France).

*Stab agar tubes*: tubes were filled with tryptose-phosphate-bouillon and aspirated with a plastic pipette (stirring the agar to dissolve it a bit) and poured into a tryptose-phosphate-bouillon tube, incubated over night at 37 °C and on the next day spread on a COS (Biomerieux, France) agar plate.

### 2.3 DNA extraction and whole genome sequencing (WGS)

A subset comprising 64 of the oldest isolates in the collection was selected and successfully recultivated. The selected *L. monocytogenes* isolates were re-sequenced using both short-read and long-read sequencing technologies in the laboratories of the Austrian Agency for Health and Food Safety (AGES), to generate high-quality hybrid assemblies and ensure accurate reconstruction of their genomic structures.

For WGS, bacterial isolates were incubated for 24 h at 37 °C under aerobic conditions on COS agar plates (Biomerieux, France) supplemented with 5% sheep blood. High-molecular weight DNA was extracted from overnight cultures using the MagAttract HMW DNA Kit (Qiagen, Germany; REF. 67563) for short-read Illumina sequencing and the NucleoSpin Microbial DNA Mini Kit (Macherey-Nagel, Germany; REF. 740235) for long-read Oxford Nanopore sequencing, following the manufacturer’s instructions for Gram-positive bacteria.

DNA was quantified using Dropsense 16 (Trinean NV/SA, Gentbrugge, Belgium) and Qubit Flex Fluorometer (Thermofisher, Austria) with 1× dsDNA High Sensivity (HS) kit (Thermofisher, Austria). For short-read sequencing, genomic libraries were prepared using the Nextera XT DNA Library Preparation kit (Illumina, San Diego, United States; FC-131-1096), following the manufacturer’s instructions. Paired-end sequencing was performed on a Miseq instrument using the MiSeq Reagent Kit v3 with a read length of 2 × 301 bp aiming for a minimum coverage of 30-fold. For long-read sequencing, genomic libraries were prepared using the Rapid Barcoding kit v14 (Oxford Nanopore Technologies, London, United Kingdom; SQK-RBK114.96) aiming for a minimum coverage of 70-fold, following the manufacturer’s instructions. Sequencing was carried out on a GridION device using FLO-MIN114 SpotON flow cells (R10.4.1).

### 2.4 Sequence data and orthogroups analysis

For short-read sequence data analysis, raw reads were *de novo* assembled using SPAdes (version 3.11.1) (Bankevich et al., 2012). Contigs were subsequently filtered using SeqSphere+ software (v10.0.2; Ridom, Münster, Germany), applying a minimum coverage threshold of 5 and a minimum contig length of 200 base pairs. For long-read sequence data analysis, nanopore reads were basecalled using Dorado v7.1.4 in superior base-calling mode and filtered using Filtlong v0.2.1^1^ with the following parameters: min_length, 1,000; keep percent, 90; and target_bases, 500,000,000. Assembly was performed using Flye v2.9.2-b1794 (Kolmogorov et al., 2019), followed by polishing with Medaka v1.8.0. For downstream hybrid assembly analysis, quality-controlled Illumina reads were used without trimming, and combined with the Nanopore data using Unicycler (v0.5.0; https://github.com/rrwick/Unicycler).

Isolate subtyping was performed using SeqSphere+ software v10.0.2 applying classical multilocus sequence typing (MLST) based on seven housekeeping genes as described by Salcedo et al. (2003), clonal complex (CC) (Ragon et al., 2008) and core genome (cg) MLST (Werner et al., 2015) analysis. Cluster threshold was set at 10 allelic differences. Serogroup information was inferred from genome data using the 5-plex PCR targets (*lmo118*, *lmo0737*, *ORF2110*, *ORF2829* and *prs*) as described (Michel et al., 2004; Lee et al., 2012), implemented in SeqSphere+ v10.0.2.

Antimicrobial resistance genes (ARGs) were identified using NCBI AMRFinder+ v3.11.2. applying the EXACT method with a 100% identity threshold. Additionally, BLAST alignment was performed against the AMRFinder+ database, using a minimum of 90% identity across protein sequences (Michael et al., 2019). Virulence genes (VGs) were identified using the Virulence Factor Database (VFDB)^2^ (Liu et al., 2022). The target scanning procedure was performed with thresholds set at ≥85% identity to the reference sequence and ≥99% identity across the aligned reference sequence. The pathogenicity islands LIPI-3 and LIPI-4 were identified using *in-house* task templates implemented and executed in SeqSphere+ v10.0.2. For LIPI-3, the presence or absence of the genes *llsA*, *llsB*, *llsD*, *llsG*, *llsH*, *llsP*, *llsX*, and *llsY* was assessed collectively to determine whether the island was present. Likewise, for LIPI-4, the combined presence or absence of *lm4b_02324*, *lm4b_02325*, *lm4b_02326*, *lm4b_02327*, *lm4b_02328*, and *lm4b_02329* was evaluated. Mobile genetic elements (MGEs) were identified using CGE Mobile Element Finder (v1.0.5), using the threshold of >90% sequence identity and ≥95% alignment to reference elements (Johansson et al., 2021). *sigB* detection was confirmed independently of the annotation-based call. The SigB protein sequence of *L. monocytogenes* EGD-e (NCBI accession NP_464421.1), an isolate not included in this collection, was retrieved from NCBI and searched by BLASTp against the PGAP-derived protein set of each of the 64 genomes. A genome was scored positive where the best hit exceeded 90% amino-acid identity over at least 90% of the query length.

Plasmid detection was performed using Chromosome & Plasmid Finder (v1.0) integrated within MOB-suite (v3.1.4), available in SeqSphere+ v10.0.2. A Mash Neighbor Distance threshold of >0.06 was used to identify plasmid sequences (Robertson and Nash, 2018). Genome annotation was performed using the Prokaryotic Genome Annotation Pipeline (PGAP) (Li et al., 2021). Orthologous groups (orthogroups) were inferred with OrthoFinder v3.0.1b1 (Emms and Kelly, 2019) in protein mode, using DIAMOND v2.2.5 (Buchfink et al., 2021) for sequence similarities search, MCL v22.282 (Enright et al., 2002) for clustering and FastME v2.1.6.3 (Lefort et al., 2015) for species-tree reconstruction. All orthogroup counts reported correspond to the hierarchical orthogroups resolved at the root node (N0) of the OrthoFinder species tree. Orthogroups present in all 64 isolates were classified as core; those detected in 2 to 63 isolates were classified as accessory; and those restricted to a single isolate were designated isolate-specific. A parallel Orthofinder DNA-mode analysis was performed as a sensitivity assessment and yielded concordant results. Orthogroups were inferred independently for the complete 64-isolate dataset. Because orthogroup membership is defined relative to the set of genomes included, counts derived from the two isolate sets are not directly comparable and are therefore reported separately.

The core-genome phylogeny corresponded to the species tree generated by OrthoFinder, which is inferred from a concatenated alignment of the 2,025 orthogroups selected by the software for species-tree reconstruction. These orthogroups are defined within OrthoFinder’s legacy orthogroup namespace, whereas the 2,097 single-copy core orthogroups reported in the Results are enumerated in the hierarchical (N0) namespace used throughout this analysis. Because the legacy and hierarchical namespaces represent distinct orthogroup definitions, the two counts refer to different entities and do not form a subset relationship.

Congruence between core-genome and accessory-genome distance was assessed using Mantel tests (Mantel, 1967). Significance was assessed by permuting isolate labels 2,999 times, with *p*-value computed as (*b* + *1*)/(*N* + 1), where *b* is the number of permutations yielding a correlation at least as extreme as that observed value (Hope, 1968); the minimum attainable *p*-value is therefore 1/3000. Labels permutation was used rather than pairs pairwise permutation because the 1,830 pairwise distances among the 61 lineage-assigned isolates derive from only 61 independent observations. Tests were performed on all isolate pairs, within each lineage, and across the lineage boundary. The between-lineage comparison forms a rectangular Lineage I by Lineage II block rather than a symmetric matrix, and was therefore treated as a Mantel-style matrix correlation in which the two margins were permuted independently, rather than as a conventional Mantel test. Because permutation-based *p*-value carry a Monte Carlo error of approximately √(*p* (1 − *p*)/*N*), about 0.005 near *p* = 0.9 at this number of permutations, non-significant values are reported to two decimal places, whereas significant values are reported as *p* < 0.001 rather than as the attainable minimum. Within-lineage trends were summarized by ordinary least-squares regression of accessory-genome Jaccard distance on core-genome patristic distance, with fitted lines drawn only across the range of core-genome distances observed in each group. Tree manipulation and patristic distances were computed in R using the ape package (Paradis and Schliep, 2019), and the Mantel and margin-permutation tests were implemented in a custom script with the random seed fixed at 1.

Each accessory orthogroup was assigned to a single functional category by keyword matching against the PGAP product description of its representative gene, using the categories transport/metabolism, prophage, transposase/integrase, restriction–modification, cell surface/internalin, virulence, stress adaptation, plasmid and antimicrobial resistance. Orthogroups whose product was annotated as a hypothetical protein, and those matching no category keyword, were recorded separately and excluded from category-level analyses. Differences in orthogroup prevalence among functional categories were tested by the Kruskal–Wallis rank-sum test (Kruskal and Wallis, 1952). For the analysis of prevalence structure, orthogroups were grouped into six prevalence bins (2, 3–5, 6–10, 11–20, 21–35 and 36–54 isolates), the upper limit corresponding to the near-core cutoff applied throughout. Independence of functional category and prevalence bin was tested by a chi-square test with Monte-Carlo estimation of the *p*-value (*B* = 9,999). Enrichment of individual category-by-bin combinations was expressed as log2 of the ratio of observed to expected counts and tested by Fisher’s exact test, with correction for multiple testing across the 48 combinations by the Benjamini–Hochberg procedure (Benjamini and Hochberg, 1995).

*L. monocytogenes* isolates belonging to each ST of our study were compared with those from pubMLST database^3^ (Moura et al., 2016) as well as the databases of the Robert Koch Institute (RKI) and the Austrian Reference Laboratory for Listeria, which currently include 28,734 genomes.

## 3 Results

### 3.1 Recultivation of *L. monocytogenes*

From the 6,451 strains included in the original “Seeliger Special Listeria Collection,” 64 *L. monocytogenes* isolates were re-cultured. Strains originated in Europe (multiple countries) (*n* = 34) and North America (*n* = 17), while the rest of isolates were isolated from unknown countries (*n* = 13) detailed in Figure 1 and Supplementary Table 1. The majority were of human origin, particularly from cerebrospinal fluid (Supplementary Table 1). Metadata for some strains have been lost and are no longer available, which limits the ability to establish epidemiological links between isolates.

**Figure 1:**
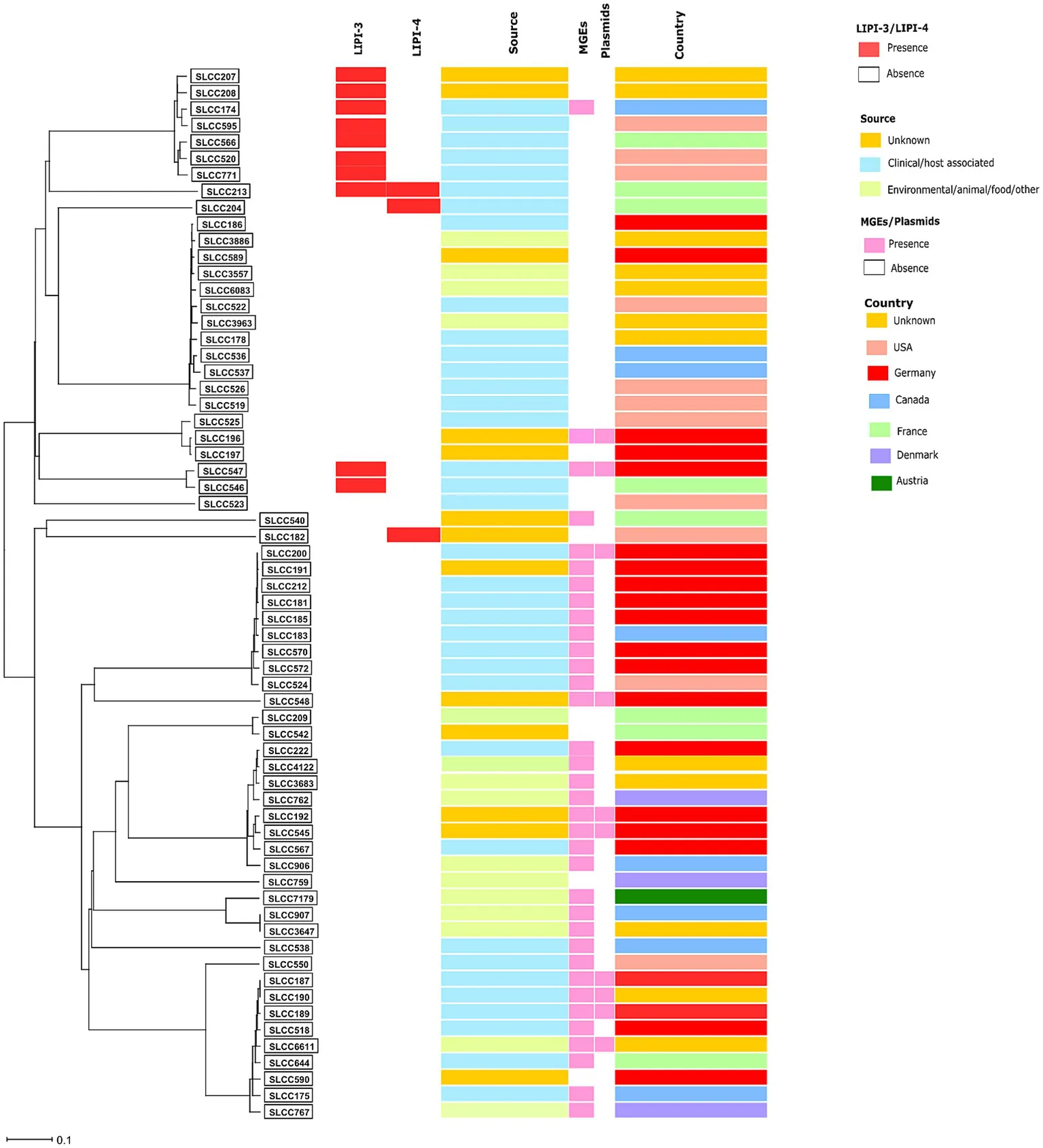
Neighbor-joining (NJ) tree showing the distribution of *L. monocytogenes* pathogenicity islands (LIPI-3 and LIPI-4), sources, mobile genetic elements (MGEs), plasmids, and countries of origin. Distance calculation is based on cgMLST scheme (1,701 targets) defined by Werner et al. (2015).

### 3.2 Whole genome sequence based subtyping, description of clusters and description of serogroups

The 64 *L. monocytogenes* isolates were distributed across five different serogroups (IIa (*n* = 28), IIb (*n* = 5), IIc (*n* = 7), IVb (*n* = 21) and L (*n* = 3)), 15 different clonal complexes (CCs), 40 different sequence types (STs) and 59 cgMLST complex types (CTs). Fourteen isolates belonged to a total of five clusters and 50 isolates were singletons (Figure 2). The isolates originated from human (*n* = 37), environment (*n* = 8), animal (*n* = 6), food (*n* = 1), and unknown (*n* = 12) sources (Figure 1, Supplementary Table 1).

**Figure 2:**
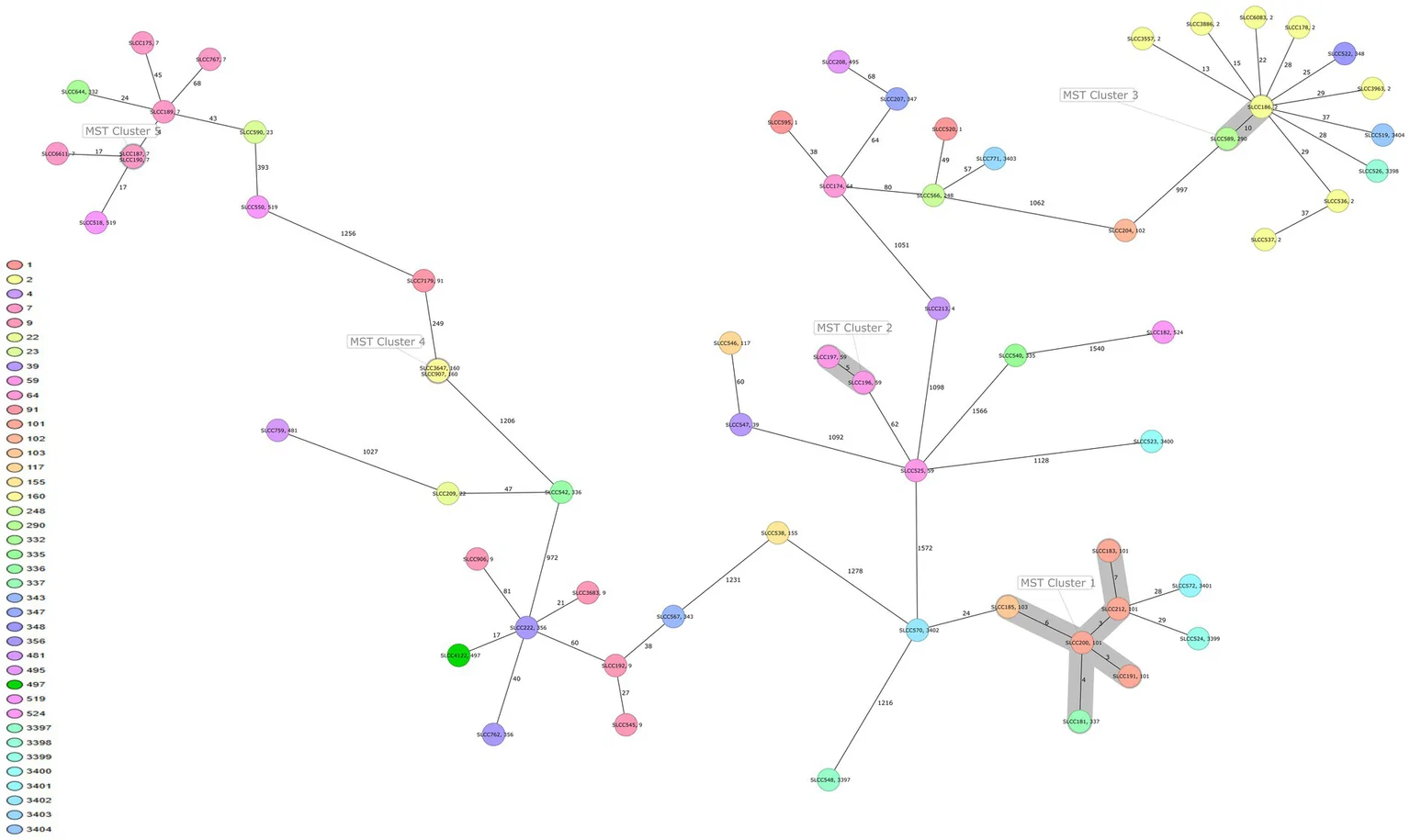
Minimum spanning tree (MST) of 64 Seeliger *L. monocytogenes* isolates analyzed in the study based on the cgMLST (1,701 targets) defined by Werner et al. (2015). Cluster definition was set in 10 allelic differences. Number on lines indicate allelic differences and numbers within nodes indicate sequence type (ST) and sample ID. Isolates are colored by ST.

*L. monocytogenes* isolates classified under serogroup IIa belonged to 18 different sequence types (STs) (Supplementary Table 1). Serogroups IIb and IIc comprised three STs, respectively (Supplementary Table 1). Isolates belonging to serogroup IVb were distributed across 13 STs (Supplementary Table 1) and serogroup L encompassed three STs (Supplementary Table 1). The most prevalent STs were ST2 (*n* = 8), ST7 (*n* = 6), ST9 (*n* = 4) and ST101 (*n* = 4), all of them retrieved between 1949–1986.

#### 3.2.1 *L. monocytogenes* ST2 (*n* = 8)

Eight *L. monocytogenes* ST2 isolates were recovered from human (*n* = 4) and environmental sources (*n* = 4). These isolates originated from Germany (*n* = 1), Canada (*n* = 2) and unspecified locations (*n* = 5) between the period 1954–1984 (Supplementary Table 1). Among them, one isolate was assigned to Cluster 3, alongside isolate SLCC589 (ST290), while remaining seven were singletons, differing by 13 to 29 alleles from isolate SLCC186 (Cluster 3) (Figure 2).

Cluster 3 included two human-derived isolates from Germany: one ST2 obtained from cerebrospinal fluid of a newborn in Hamburg in 1954 and the other ST290 from an adult in Leipzig in 1957 differing by 10 alleles from each other. Singleton SLCC3557 recovered from soil in 1972 (unknown location) showed 13 allelic differences to the Cluster 3 isolates (Figure 2). Using the core genome (1,701 gene targets) and the accessory genome for analysis (1,158 gene targets), the two isolates (SLCC186 and SLCC589) differed by 15 alleles (Figure 3).

**Figure 3:**
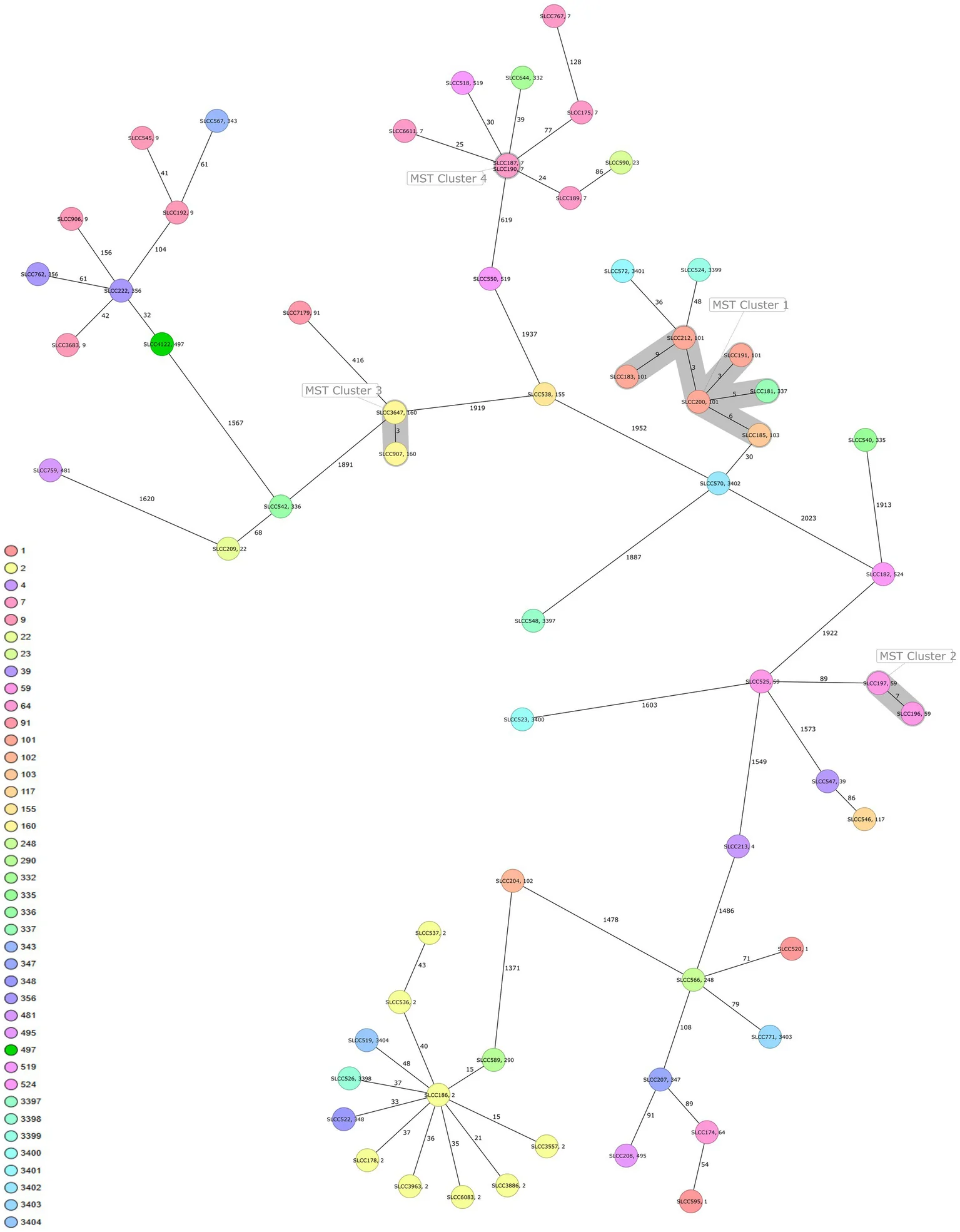
Minimum spanning tree (MST) of 64 Seeliger *L. monocytogenes* isolates analyzed in the study based on the cgMLST (1,701 targets) and accessory (1,158 targets) schemes defined by Werner et al. (2015). Cluster definition was set in 10 allelic differences. Number on lines indicate allelic differences and numbers within nodes indicate sequence type (ST) and sample ID. Isolates are colored by ST.

The environmental ST2 *L. monocytogenes* singletons (*n* = 4) were retrieved from soil (1972 and 1973), hay (1973) and sewage (1984), and differed by 21 to 38 alleles from each other. The human ST2 *L. monocytogenes* singletons (*n* = 3) were isolated from the blood of a newborn and the vagina of a mother in Canada (1957) differing by 37 alleles. The isolate obtained from cerebrospinal fluid in 1954 with unknown location (SLCC178), differed by 61 alleles from the Canadian newborn (SLCC536) and mother (SLCC537) isolates (Figure 4).

**Figure 4:**
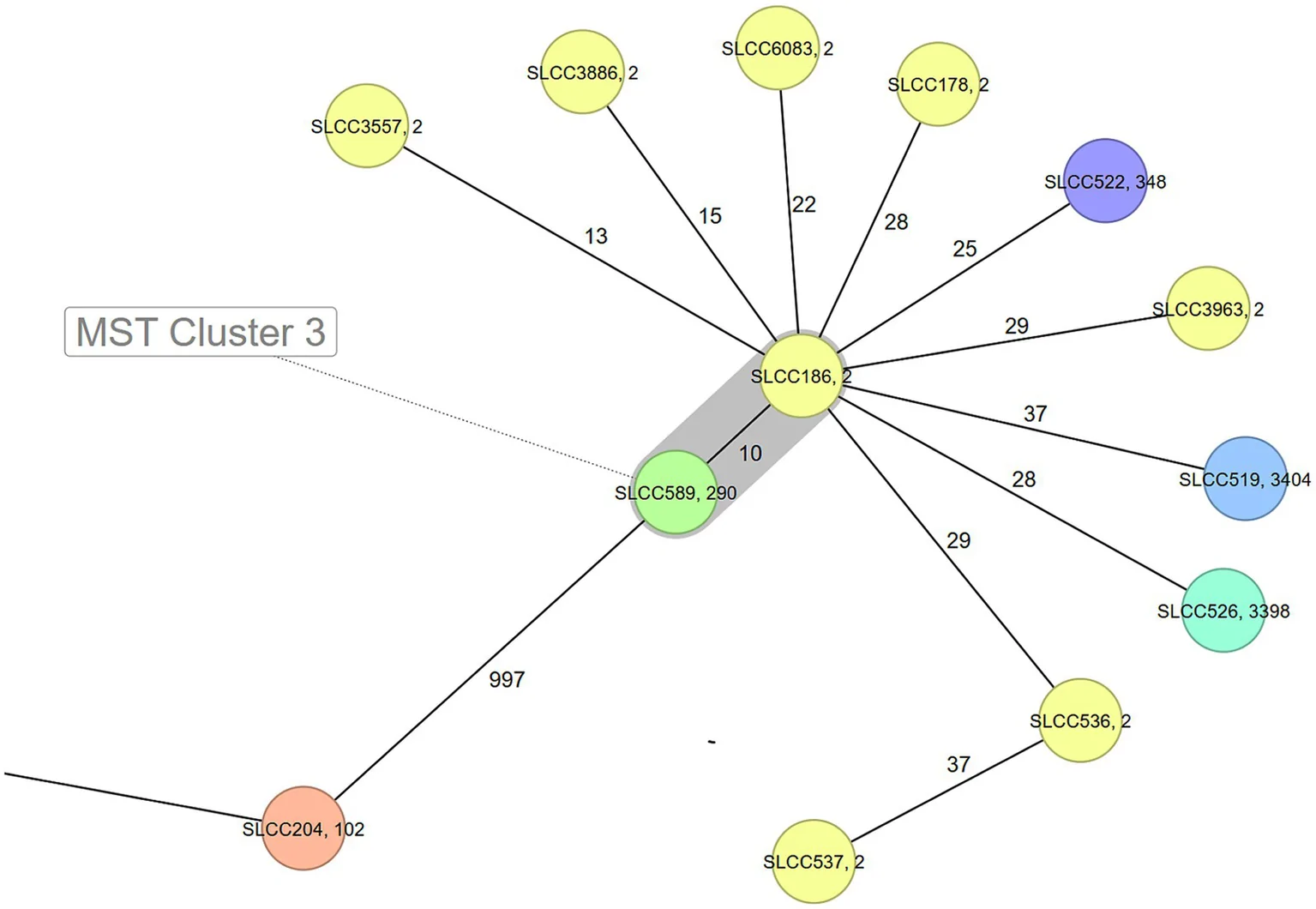
Zoom-in of MST Cluster 3 formed by two isolates (SLCC186, ST2 and SLCC589, ST290).

#### 3.2.2 *L. monocytogenes* ST7 (*n* = 6)

Six *L. monocytogenes* ST7 isolates were recovered from human (*n* = 4), animal (*n* = 1) and environmental sources (*n* = 1). These isolates originated from Germany (*n* = 2), Canada (*n* = 1), Denmark (*n* = 1) and unknown location (*n* = 2) between 1953–1986 (Supplementary Table 1).

Two isolates (SLCC187 and SLCC190) belonged to the same CT1198 (Cluster 5): strain SLCC187 was retrieved from cerebrospinal fluid of an adult with meningitis in Moers in 1954, strain SLCC190 was isolated from a clinical sample (unknown year and country) (Figures 2, 5). Core genome (1,701 gene targets) and accessory genome analysis (1,158 gene targets), showed again no allelic differences (Cluster 4 in Figure 3).

**Figure 5:**
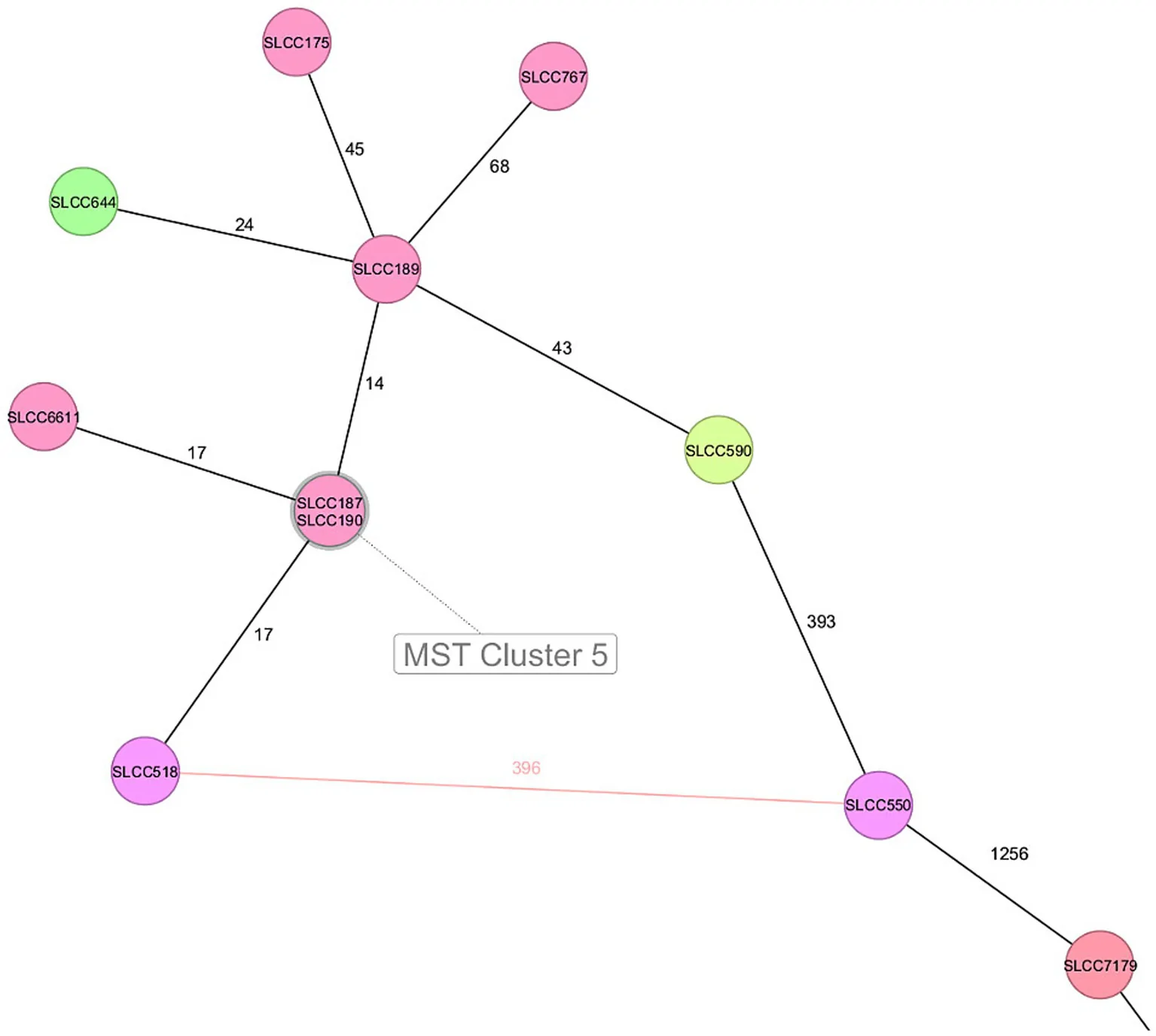
Zoom-in of MST Cluster 5 formed by two Seeliger *L. monocytogenes* isolates (SLCC187 and SLCC190, both ST7) and allelic differences between the two Seeliger *L. monocytogenes* isolates ST519 (SLCC550 and SLCC518).

Four ST7 *L. monocytogenes* singletons (SLCC175, SLCC189, SLCC767, SLCC6611) were recovered between 1953 and 1986 from diverse sources: cow placenta in Denmark, soil from unspecified location, cerebrospinal fluid from a child in Canada, and cerebrospinal fluid from an adult in Stralsund (Germany) (Supplementary Table 1). These singletons differed by a minimum of 14 alleles from Cluster 5 (Figure 5).

ST7 *L. monocytogenes* isolates assigned to Cluster 5 (Figure 2) grouped with three Seeliger *L. monocytogenes* isolates retrieved from pubMLST database (see text footnote 3): Isolates SLCC307 and SLCC309 were retrieved from newborns from Greifswald, Germany in 1953; and isolate SLCC2653 was retrieved from a 61-years-old female with meningitis in Mainz, Germany in 1967 (Supplementary Table 1). These isolates, part of Cluster 2 in Figure 6, differed by 7–10 alleles from each other.

**Figure 6:**
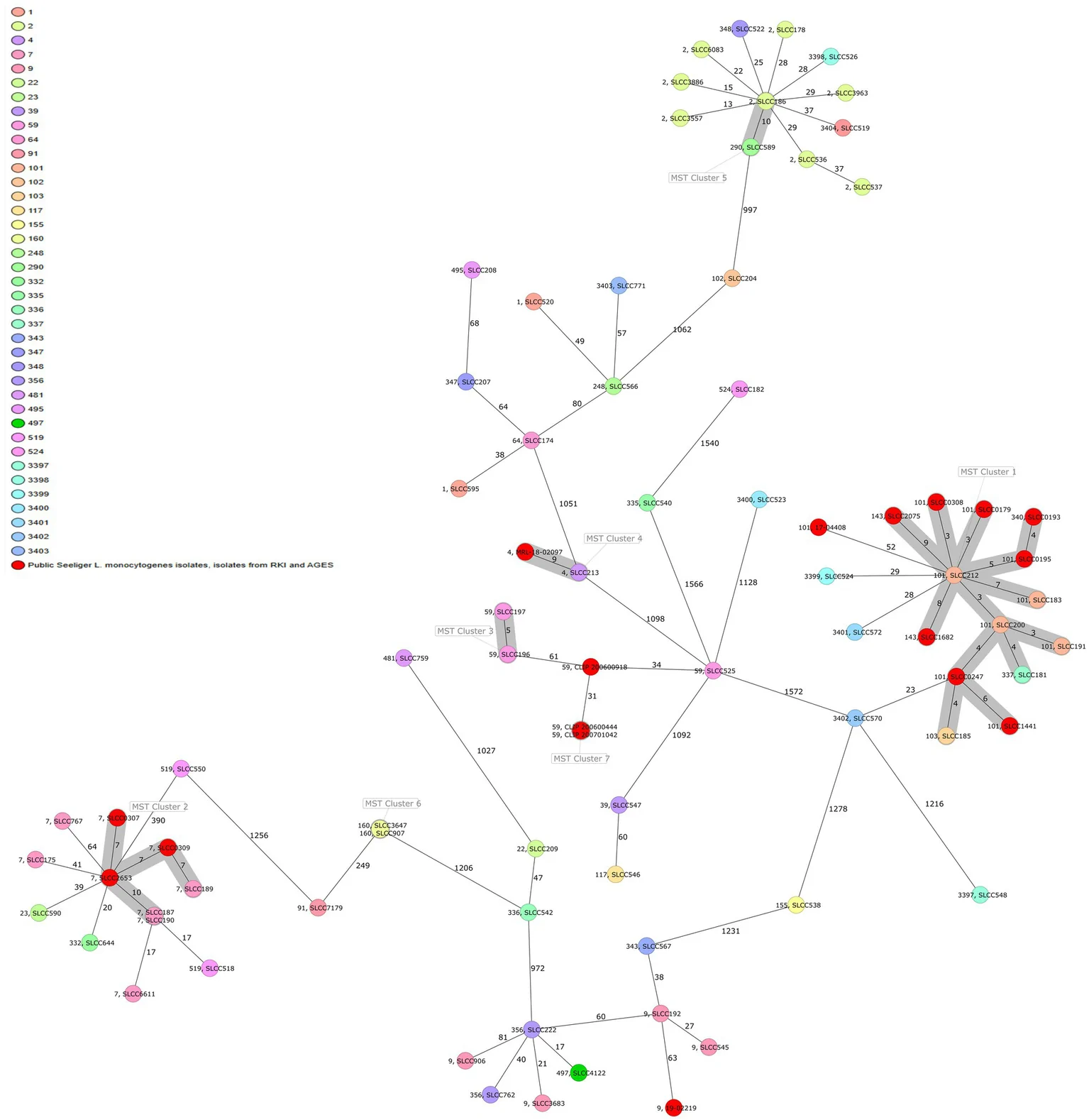
Minimum spanning tree (MST) of 64 Seeliger *L. monocytogenes* isolates analyzed in the study based on the cgMLST (1,701 targets) defined by Werner et al. (2015). Cluster definition was set in 10 allelic differences. Number on lines indicate allelic differences and numbers within nodes indicate sequence type (ST) and sample ID. Isolates are colored by ST. Red nodes indicate Seeliger and additional *L. monocytogenes* isolates available in public and from internal database (Robert Koch Institute, Germany; AGES, Austria).

#### 3.2.3 *L. monocytogenes* ST9 (*n* = 4)

Four *L. monocytogenes* ST9 singletons were recovered from diverse sources: liver of a canary in Canada (1949), fir needle from unspecified location (1972), human in Leipzig (Germany) and the brain of a patient with meningitis in Berlin (Germany) in 1957 (Figure 2, Supplementary Table 1). These isolates exhibited 67 to 99 allelic differences among themselves. Furthermore, ST9 isolates differed by 21 to 81 alleles from singleton SLCC222 (ST356), which was recovered from a newborn in Halle (Germany) in 1955 (Figure 2, Supplementary Table 1). Database search revealed that SLCC192 differs by 63 alleles from a recent *L. monocytogenes* isolate 19–02219 (isolated in Germany in 2019 from a human case of listeriosis) from the RKI internal database.

#### 3.2.4 *L. monocytogenes* ST101 (*n* = 4) and cluster 1

Four *L. monocytogenes* ST101 isolates belonged to Cluster 1 along with SLCC181 (ST337) and SLCC185 (ST103), differing by 3 to 7 alleles (Figure 2). When analyzing the data with cgMLST (1,701 gene targets) and the accessory genome set (1,158 gene targets), the composition of Cluster 1 remained unchanged, although minor changes (allelic differences were slightly higher, ranging from 3 to 9 alleles) were observed in their allelic differences (Figure 3).

*L. monocytogenes* isolates from Cluster 1, involving three different STs (ST101, ST337 and ST103), were all recovered from diverse human sources: SLCC183 from the vagina of a mother in Canada, SLCC191 from an unspecified source in Halle (Germany), SLCC200 from a newborn in Halle (Germany), and SLCC212 from a premature born in Göttingen (Germany), SLCC181 (ST337) from meconium in Halle (Germany), and SLCC185 (ST103) from a man (unknown sample material) in Leipzig (Germany) (all isolates from 1954) (Figure 2, Supplementary Table 1).

The isolates from Cluster 1 (previously described comprising six distinct isolates with different STs in Figure 2) clustered with other Seeliger *L. monocytogenes* isolate-patterns retrieved from the pubMLST database (see text footnote 3). Specifically, they clustered with a total of eight Seeliger *L. monocytogenes* isolates; five ST101 Seeliger *L. monocytogenes* isolates (SLCC0247, SLCC0179, SLCC0308, SLCC0195, SLCC1441) recovered from human (females in all cases but one unspecified) between 1953 and 1962 in different cities from Germany (Hannover, Leipzig, Greifswald and Magdeburg); two ST143 *L. monocytogenes* isolates from human cases in Leipzig and Braunschweig (Germany) in 1964 and an unspecified year; and one ST340 *L. monocytogenes* isolate from a human case in Magdeburg (Germany) in 1954 (Cluster 1; Figure 6, Supplementary Table 1). The German *L. monocytogenes* isolate 17-04408 (ST101, pattern retrieved from the RKI internal database; isolated from Germany from a human case of listeriosis in 2017) differed by 52 alleles from isolate SLCC212 (ST101) within Cluster 1 (Figure 6).

### 3.3 Less prevalent STs

#### 3.3.1 *L. monocytogenes* ST1 (*n* = 2)

Two *L. monocytogenes* ST1 isolates (SLCC520 and SLCC595) were recovered from human cerebrospinal fluid samples in the United States between 1956 and 1957, differing from each other by 98 alleles. Their closest genetic relatives were SLCC566 (ST248), isolated from a vaginal sample in Toronto (Canada) in 1953, and SLCC174 (ST64), obtained from cerebrospinal fluid of a meningitis patient in Lyon (France) in 1957, differing by 38–49 alleles. All isolates belonged to CC1, together with singletons SLCC207 (ST347) and SLCC208 (ST495) (Figure 2, Supplementary Table 1).

#### 3.3.2 *L. monocytogenes* ST4 (*n* = 1)

One *L. monocytogenes* ST4 singleton (SLCC213) was not closely related to any other *L. monocytogenes* isolate (the closest was singleton SLCC174, ST64 with 1,051 allelic differences) (Figure 2). SLCC213 was recovered from a newborn in Paris in 1954 (Supplementary Table 1). Nevertheless, *L. monocytogenes* SLCC213 clustered with *L. monocytogenes* MRL-18-02097 (ST4, isolated from compost in Austria, 2018, pattern retrieved from AGES internal database) (Cluster 4 in Figure 6) (Supplementary Table 1). Cluster 4 showed no genetic relatedness to any other cluster or singleton.

#### 3.3.3 *L. monocytogenes* ST59 (*n* = 3)

Cluster 2 of *L. monocytogenes* comprised two isolates (SLCC196 and SLCC197), both recovered from human cases in 1954 in Wernigerode and Leipzig (Germany), with five allelic differences (Figure 2). This cluster differed by 62 alleles from the singleton SLCC525 (ST59), which was obtained from cerebrospinal fluid in 1956 in New Orleans, United States (Figure 2, Supplementary Table 1). Using cgMLST combined with accessory genome analysis (1,158 gene targets), the isolates assigned to Cluster 2 remained consistent; however, seven allelic differences were observed (Figure 3).

Comparison to ST59 isolates from the pubMLST database (see text footnote 3) revealed 31–61 allelic differences (Figure 6).

#### 3.3.4 *L. monocytogenes* ST160 (*n* = 2)

Two *L. monocytogenes* ST160 isolates (SLCC907 and SLCC3647) formed Cluster 4 with no allelic differences among them (Figure 2). SLCC3647 was retrieved from soil of unspecified location in 1972, and SLCC907 from a lemming in Canada in 1953 (Supplementary Table 1). They were not closely related to any other *L. monocytogenes* isolate in our study (the closest was an ST91 isolate from cheese in Austria (1986), which was 249 alleles distant). Using cgMLST combined with accessory genome analysis (1,158 gene targets), the isolates assigned to Cluster 4 differed by three alleles (Cluster 3, Figure 3).

#### 3.3.5 *L. monocytogenes* ST356 (*n* = 2)

Two *L. monocytogenes* ST356 isolates (SLCC222 and SLCC762) differed 39 alleles from each other (Figure 2). SLCC762 was retrieved from a silver fox (*Vulpes vulpes morfa argentea*) in Denmark in 1941 and SLCC222 from a newborn in Germany (Halle) in 1955 (Supplementary Table 1).

#### 3.3.6 *L. monocytogenes* ST519 (*n* = 2)

Two *L. monocytogenes* ST519 isolates (SLCC518 and SLCC550) differed by 396 alleles (Figures 2, 5). Both isolates were recovered from cerebrospinal fluid - one from a patient with meningitis—in two different locations: New York (USA) and Gelsenkirchen (Germany), both in 1957 (Supplementary Table 1).

### 3.4 Other STs

The *L. monocytogenes* singleton SLCC182 (ST524) was not closely related to any isolate in this study; its nearest neighbor was SLCC540 (ST335), with 1,540 allelic differences (Figure 2). Similarly, *L. monocytogenes* isolate SLCC548 (ST3397) showed no close relatedness to other isolates; the closest was SLCC570 (ST3402) with 1,216 allelic differences (Figure 2). *L. monocytogenes* isolate SLCC526 (ST3398) presented 28 allelic differences from its nearest isolate, SLCC186 (ST2) (Figure 2). SLCC523 (ST3400) was also distantly related to all other isolates; the closest was SLCC525 (ST59) with 1,128 allelic differences (Figure 2). SLCC524 (ST3399) and SLCC572 (ST3401) differed by 28-29 alleles from SLCC212 (ST101, Cluster 1) (Figure 2). SLCC771 (ST3403) differed by 57 alleles from singleton SLCC566 (ST248). Finally, singleton SLCC519 (ST3404) differed by 37 alleles from SLCC186 (ST2, Cluster 4) (Figure 2).

### 3.5 Mobile genetic elements (MGEs) and plasmids

Thirty-five out of 64 isolates contained at least one MGE, and nine *L. monocytogenes* isolates harbored more than five MGEs. The most prevalent MGEs were the unit transposon *ISLmo1*, detected in 33 *L. monocytogenes* isolates, followed by the transposon *Tn5422* detected in 12 *L. monocytogenes* isolates and the insertion sequence *ISLmo9* and *ISLmo4,* found in eleven and nine *L. monocytogenes* isolates, respectively (Figure 1, Supplementary Table 1).

A *L. monocytogenes* belonging to ST7, ST9 and ST101 (among the most prevalent STs) carried at least one MGE. Among the less prevalent STs, *L. monocytogenes* ST59, ST160 (*n* = 2), ST356 (*n* = 2) and ST519 (*n* = 2) carried at least one MGEs. Additional STs, such as (*n* = 1) ST155, ST335, ST39, ST3397, ST3399, ST3401, ST3402, ST332, ST343, ST337, ST91, ST64, ST103 and ST497 carried MGEs (Supplementary Table 1). On the other hand, *L. monocytogenes* isolates ST1, ST2, ST4, ST22, ST23, ST102, ST117, ST248, ST290, ST336, ST347, ST348, ST481, ST495, ST524, ST3398, ST3400, ST3403 did not carry MGEs (Supplementary Table 1).

Ten out of 64 *L. monocytogenes* isolates harbored one plasmid (Figure 1, Supplementary Table 1), whose size ranged from 45.2–432 kb. Four ST7 *L. monocytogenes* isolates, two ST9 *L. monocytogenes* isolates, and one isolate each from ST39, ST59, ST101 and ST3397 carried a plasmid (Supplementary Table 1). Those STs harboring plasmids tended to possess the highest number of MGEs as well. Additionally, ST7 isolates carried both MGEs and plasmids simultaneously (Supplementary Table 1). These ST7 isolates originated from diverse sources: bovine (cow, placenta), environment (soil), human (cerebrospinal fluid from meningitis cases and of unknown sources) and were collected between 1954 and 1986 across Denmark, Canada and Germany (Halle, Moers and Stralsund) (Supplementary Table 1).

### 3.6 Antimicrobial resistance genes (ARGs), stress-related gene *sigB*, pathogenicity islands and virulence genes (VGs)

The *fosX* and *lin* genes, which confer resistance to fosfomycin and lincosamide, respectively, were detected in all 64 *L. monocytogenes* isolates analyzed in this study. The stress-related gene *sigB* was present in all 64 *L. monocytogenes* isolates included in our study (Supplementary Table 1). Detection of *sigB* by sequence homology agreed completely with the annotation-based call. The EGD-e SigB protein was recovered from all 64 genomes at 99.2 to 100% amino-acid identity over 100% of the query length (*E* = 0.0; bit scores 525 to 529), with no disagreement between the two approaches. The gene is therefore present and full-length throughout the collection, and the two independent lines of evidence together exclude a threshold or template artifact in its detection. Additionally, all known *Listeria* VG variants were detected in the collection, although not all were present in every individual isolate. The following VGs were present in all 64 analyzed *L. monocytogenes* isolates: *bsh*, *clpC*, *clpE*, *clpP*, *fbpA*, *hyl*, *hpt*, *inlC*, *lap*, *lntA*, *lpeA*, *lspA*, *mpl*, *plcA*, *plcB*, *prfA* and *prsA2* (Supplementary Table 1).

Eleven out of 64 *L. monocytogenes* isolates carried pathogenicity island LIPI-3 (Figure 1, Supplementary Table 1). These included isolates from clinical and environmental sources belonging to different STs detailed in Supplementary Table 1. One *L. monocytogenes* ST39 isolate carrying LIPI-3 also harbored multiple MGEs and one plasmid simultaneously. Three out of 64 *L. monocytogenes* isolates carried pathogenicity island LIPI-4 (Figure 1, Supplementary Table 1). These included isolates from clinical and unknown sources between 1950 and 1954 from different STs detailed in Supplementary Table 1. *L. monocytogenes* isolate SLCC213 (ST4)—harboring LIPI-3 and LIPI-4 simultaneously—was obtained from a newborn in 1954 (France). LIPI-1 was assessed as an island rather than as a set of independent genes, by requiring the six constituent genes *prfA*, *plcA*, *hly*, *mpl*, *actA* and *plcB* to be present, physically linked on a single contig and annotated without disruption. The island was intact in 63 of the 64 isolates. In SLCC644 all six genes were present and contiguous across a span of 8,654 bp, but *prfA* was annotated as a pseudogene, indicating a truncated or frameshifted copy of the master virulence regulator rather than loss of the island. Because SLCC644 is assembled at contig level, this disruption requires confirmation by direct inspection of the underlying sequence before it is interpreted as genuine, since frameshifts arising from assembly or basecalling error cannot be excluded from a draft assembly.

### 3.7 Presence of orthogroups in *L. monocytogenes* strains

Orthogroups were inferred for the complete *L. monocytogenes* dataset using both protein-level and coding-sequence-level analyses (Table 1). The total number of orthogroups proportion belonging to the core, was 57.3% in protein mode and 52.9% in DNA mode.

**Table 1:** Orthougroups composition across the analysis.

Analysis	Total	Core	Single-copy	Accessory	Isolate-specific
64 isolates protein	3,717	2,129 (57.3%)	2,097	1,582 (42.6%)	6
64 isolates DNA	3,936	2,084 (52.9%)	2,078	1,845 (46.9%)	7

Percentages are of the total in that analysis.

Across the 64 genomes, 3,717 orthogroups were delineated, including 2,129 (57.3%) present in all isolates (constituting the core)—of which 2,097 were single-copy core orthogroups. A further 1,582 orthogroups (42.6%) were shared by subsets of 2 to 63 isolates, and only 6 were isolate-specific. Additionally, 359 genes were not assigned to any orthogroup. Accessory-genome content ranged between 580 to 827 orthogroups per isolate (mean 689). These patterns indicate that non-core variation is predominantly shared among multiple isolates rather than restricted to individual genomes.

Pairwise accessory-genome Jaccard distances ranged from 0.003 to 0.595 (mean 0.378). Ordering the distance matrix according to the core-genome phylogeny resolved two blocks of low internal distance separated by a block of uniformly high distance (Figure 7). These corresponded precisely to the two principal lineages: the lineage-separating bipartition of the core-genome tree reproduced the serogroup-derived assignment without discordant isolates, separating 26 Lineage I isolates (21 IVb, 5 IIb) from 35 Lineage II isolates (28 IIa, 7 IIc). Isolates belonging to the same lineage shared substantially more accessory content than isolates from different lineages (Jaccard 0.282 ± 0.099 versus 0.463 ± 0.040), and approximately 59% of the variance in pairwise accessory distance was associated with lineage membership. This proportion is a descriptive measure based on non-independent pairs and does not represent a population-level effect.

**Figure 7:**
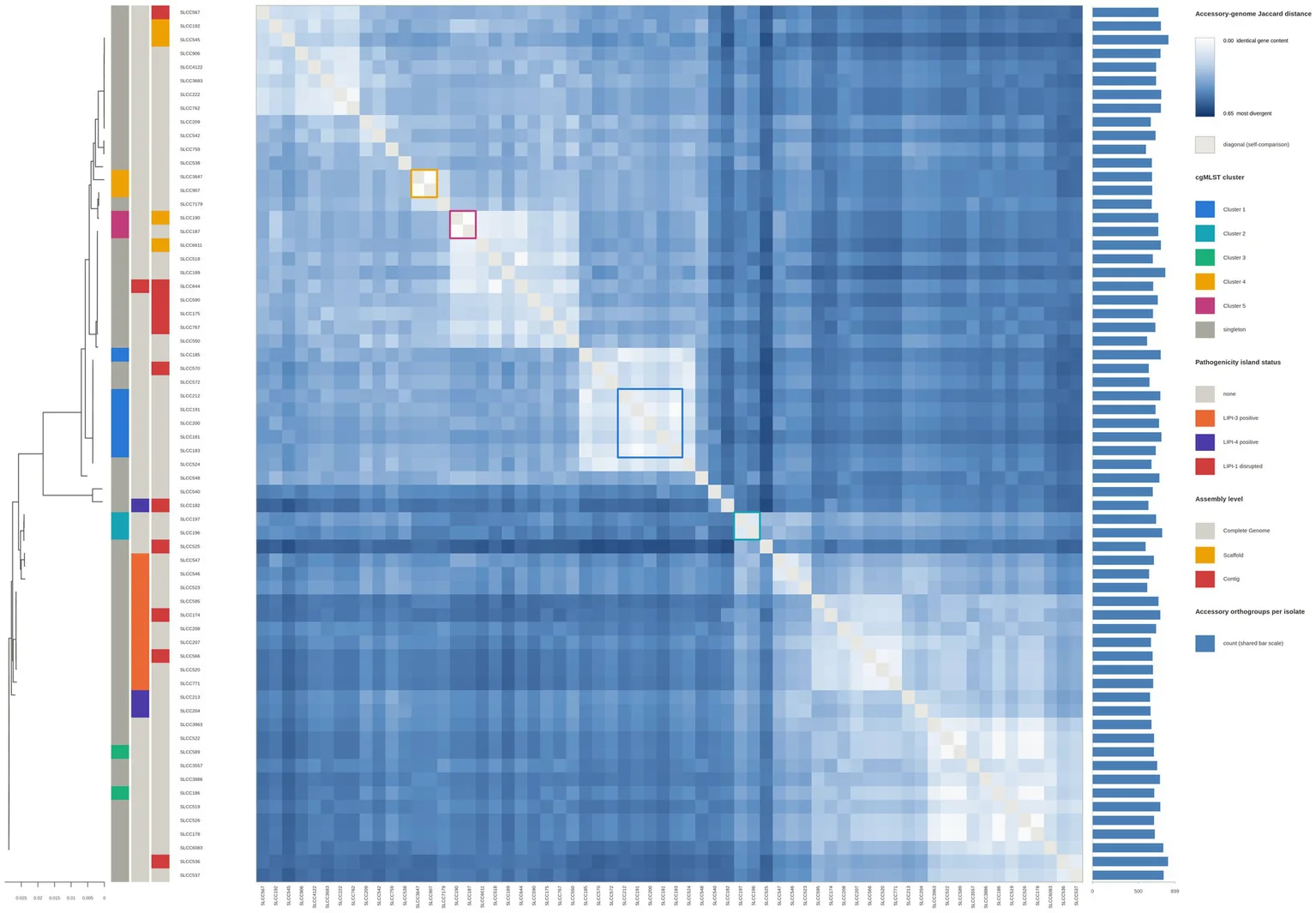
Accessory-genome Jaccard distance among the 64 *Listeria* isolates, ordered according to their core-genome phylogeny shown at left (scale bar in substitutions per site). Isolates are ordered identically on both axes. Matrix shading reflects pairwise accessory-genome Jaccard distance, from light (identical accessory content) to dark, with a display limit of 0.65 (maximum observed: 0.595). The gray diagonal marks self-comparisons. Colored strips indicate, from left to right, cgMLST clusters, pathogenicity-island status and assembly level. Outlined diagonal blocks highlight cgMLST clusters that are also phylogenetically contiguous. Bars on the right show the number of accessory orthogroups per isolate. Two low distance blocks correspond to Lineage I and Lineage II, while the uniformly dark region between them represents between-lineage comparisons.

Across all isolate pairs, accessory-genome distance was strongly correlated with core-genome patristic distance (Mantel *r* = 0.823, *p* < 0.001; Figure 8A). The distribution of core-genome distances was discontinuous, with no pairwise values falling between 0.009 and 0.022 substitution per site. Within each lineage, the correlation persisted and was slightly stronger (*r* = 0.841 in Lineage I and *r* = 0.854 in Lineage II, both *p* < 0.001; Figure 8B). In contrast, among the 910 pairs spanning the two lineages was absent (*r* = −0.003, *p* = 0.97, margin-permutation test), and accessory-genome distances remaining narrowly centered at 0.463 ± 0.040 across core-genome distances of 0.022 to 0.029 substitutions per site. Between-lineage accessory distances therefore formed an approximately constant band over the observed core-genome range, consistent with, but not sufficient to demonstrate, a saturation-like plateau, as the transition between the two regimes lies within in the unsampled interval. Both within-lineage regressions yielded positive intercepts close to 0.14, and among the 193 isolate pairs with no measurable core-genome divergence, the accessory Jaccard distance reached 0.350. Thus, even complete core-genome identity at this resolution does not imply identical accessory-genome content, with up to 35% of orthogroups in the pairwise union not shared.

**Figure 8:**
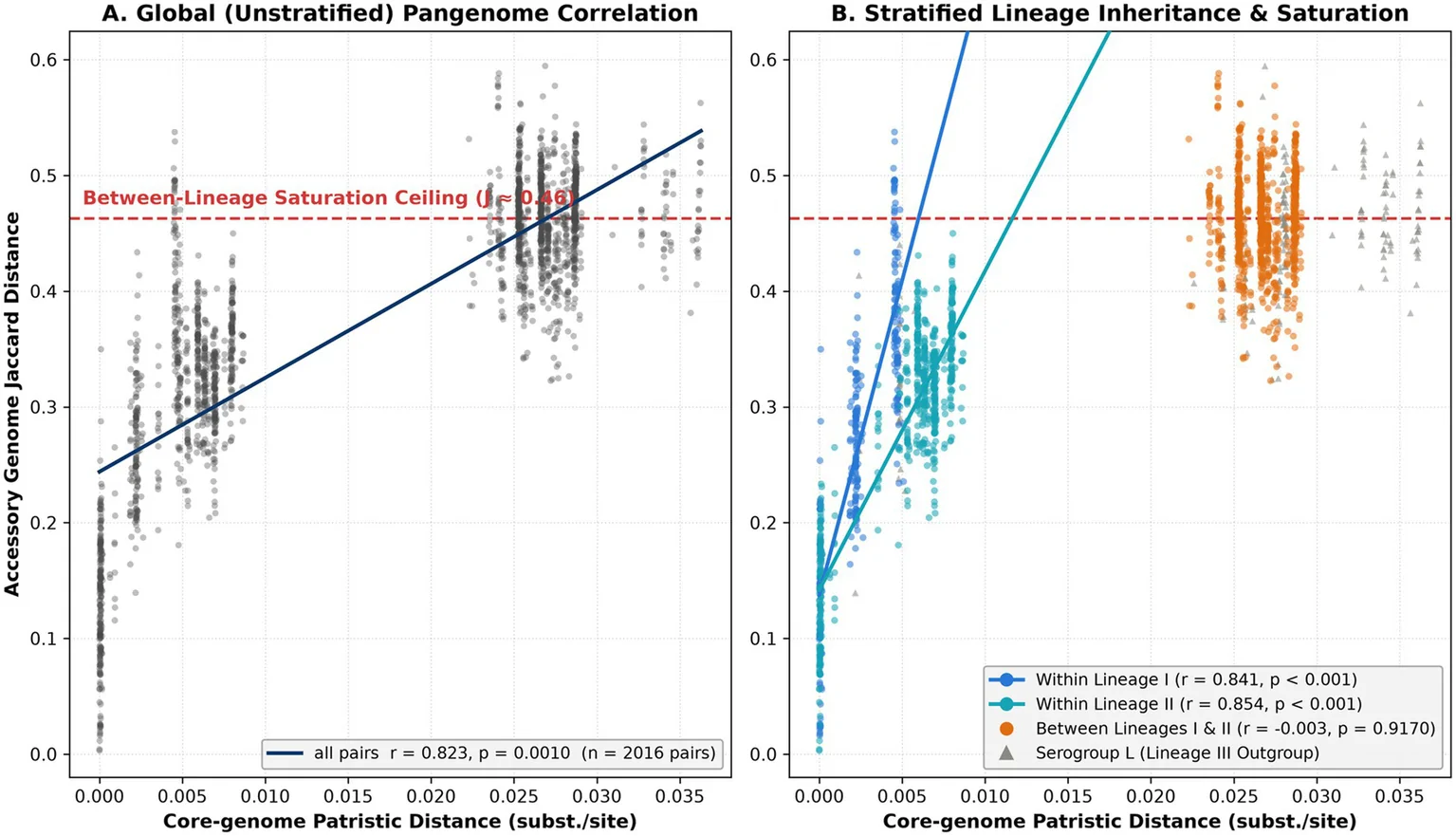
Relationship between core-genome patristic distance and accessory-genome Jaccard distance across 2,016 isolate pairs, shown globally and by lineage. **(A)** Global correlation between core and accessory divergence (Mantel *r* = 0.823, *p* < 0.001); the dashed line marks the mean between-lineage accessory distance (0.463). **(B)** Lineage-stratified patterns: Strong positive associations within Lineage I and Lineage II (Mantel *r* = 0.841 and 0.854), no association between lineages (*r* = −0.003, *p* = 0.97), and serogroup L isolates excluded from stratified tests. The gap between ~0.009 and 0.022 substitutions per site separates within-lineage from between-lineage comparisons.

A total of 1,130 accessory orthogroups were shared by 2 out of 54 isolates. From those, 251 (22.2%) were assigned to a defined functional category (Derived from Orthofinder in protein mode) and 879 were annotated as hypothetical proteins or matched no category. Prevalence differed significantly among functional categories (Kruskal–Wallis *χ*^2^ = 17.8, d*f* = 7, *p* = 0.010; Figure 9A). Plasmid-associated orthogroups were the least widespread (median prevalence 3 isolates, 4 orthogroups), followed by prophage (8, *n* = 54), transposase/integrase (9, *n* = 30), virulence (10.5, *n* = 16), restriction–modification (11, *n* = 37) and stress adaptation (12.5, *n* = 10), while transport and metabolic functions (15, *n* = 79) and cell surface and internalin functions (18, *n* = 21) were the most widely shared.

**Figure 9:**
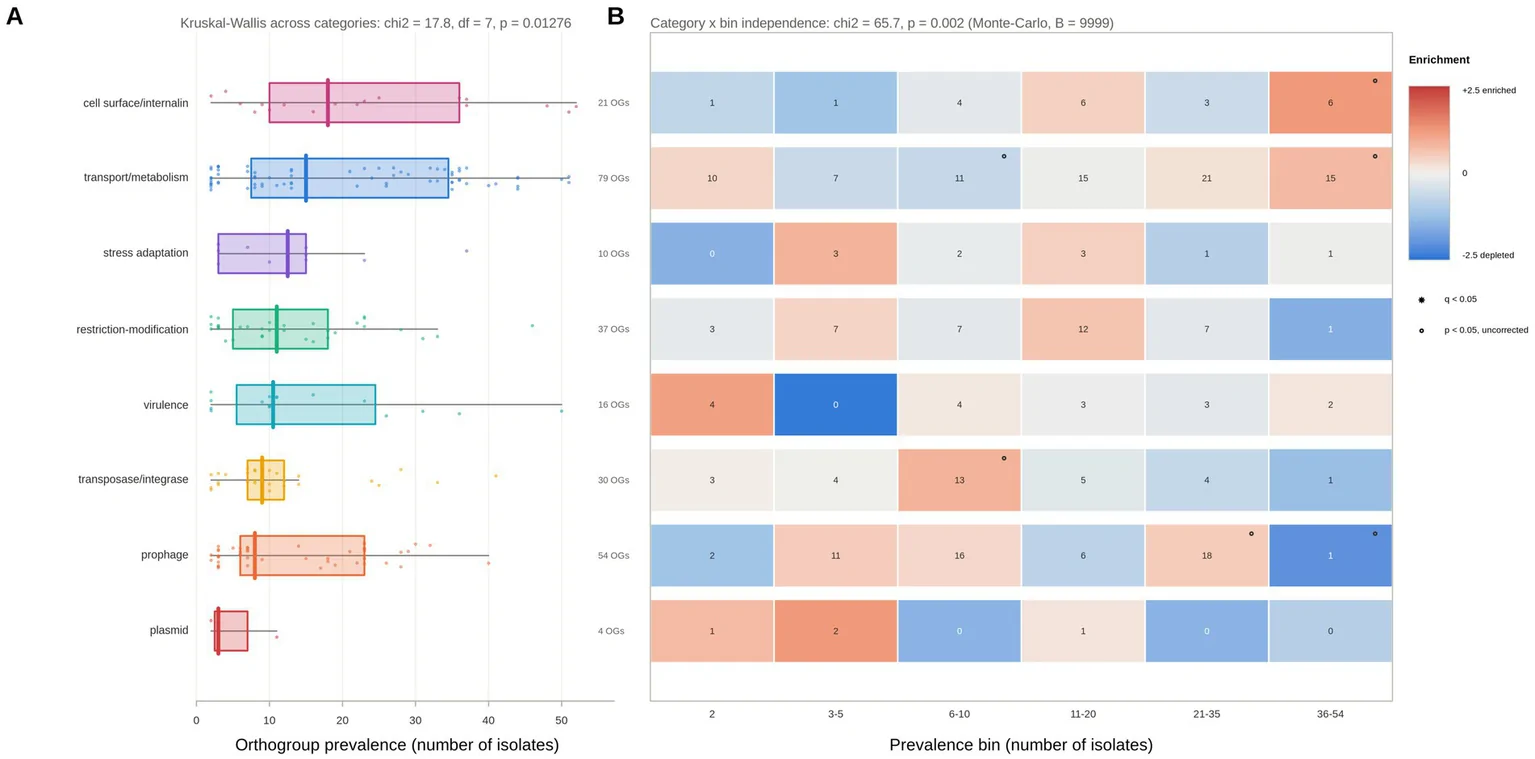
Functional composition of the accessory genome by orthogroup prevalence. **(A)** Prevalence of orthogroups within each functional category, ordered by median; boxes show the IQR, whiskers the full range, and points individual orthogroups. Counts indicate the number of orthogroups per category. Of 1,130 accessory orthogroups (present in 2–54 isolates), 251 were assigned to defined categories; the remaining 879 were hypothetical or unclassified. Prevalence differed significantly among categories (Kruskal–Wallis *χ*^2^ = 17.8, d*f* = 7, *p* = 0.010). **(B)** Enrichment of functional categories across prevalence bins, colored by log_2_ observed/expected ratios; cell values are orthogroup counts. Category and prevalence bin were not independent (*χ*^2^ = 65.7, Monte-Carlo *p* = 0.002, *B* = 9,999). No individual combinations passed FDR correction (minimum *q* = 0.185). Values derive from the 64-isolate protein-mode analysis; orthogroups present in >54 isolates were excluded as near-core.

Functional category was not independent of prevalence bin (*χ*^2^ = 65.7, Monte-Carlo *p* = 0.002; Figure 9B). The largest deviations from expectation occurred among the most widespread orthogroups, those present in 36 to 54 isolates, where prophage-associated orthogroups were under-represented (1 observed against 5.8 expected) while transport and metabolic orthogroups (15 against 8.5) and cell surface and internalin orthogroups (6 against 2.3) were over-represented; transposase and integrase orthogroups were over-represented among orthogroups present in 6 to 10 isolates (13 against 6.8). No individual category-by-bin combination remained significant after correction for multiple testing (minimum *q* = 0.185), and these per-cell differences are therefore reported descriptively.

## 4 Discussion

### 4.1 Genetic diversity

Controlling virulence traits in *L. monocytogenes* continues to be a major challenge due to this organisms remarkable resilience across different environments. Addressing these challenges requires comprehensive strain analysis that captures the ecological and temporal diversity. In this context, the Seeliger special collection represents an exceptional set of samples for studying *L. monocytogenes* from a One Health perspective. Given the professional trajectory of Professor H. P. Seeliger, who was the leading global authority on *Listeria* in the last century, he collected thousands of strains with the aim of documenting the natural diversity, establishing solid taxonomic foundations, studying its epidemiology, and he preserved isolates from a wide variety of sources over several decades. By doing so, Professor H. P. Seeliger created a unique collection that enables in-depth investigations of the evolution, dissemination and pathogenicity of *L. monocytogenes*. However, the key limitation of the study is that the analysis was conducted on a relatively small set of isolates; The isolates included in the study span the period from 1918 to 1986, originating from several European countries, as well as from Canada and the United States. Although the *L. monocytogenes* isolates included cover a broad range of years and sources, the dataset may not be representative.

WGS analysis revealed that the 64 *L. monocytogenes* isolates included in our study belonged to serogroups IIa, IIb, IIc, IVb and L. Serogroup IIa was the most prevalent (43.75%), followed closely by IVb (32.8%), while serogroup L (4a/4c and *L.* spp.) showed the lowest prevalence (4.68%). The designation of the serogroup “L” indicates that the isolate is a *Listeria*, but it cannot be classified into a specific serogroup (Doumith et al., 2005). Serogroup IVb is the most clearly associated with listeriosis outbreaks, according to the scientific literature (Magalhaes et al., 2015; Halbedel et al., 2021; Esposito et al., 2021). However, serogroup IIa, especifically *L. monocytogenes* ST155, has been linked to outbreaks associated with ready-to-eat fish products. A notable example is a long-lasting outbreak that began in Sweden related to smoked salmon and subsequently spread to other European countries, persisting for more than 10 years (EFSA, 2023). In our study, only a single *L. monocytogenes* ST155 isolate (SLCC538) was identified. This isolate was detected in a newborn in Canada in 1957 and there is no evidence that shows connection to the outbreak mentioned above.

Several of the most prevalent sequence types (STs) identified among our isolates were *Listeria monocytogenes* ST2, ST7, and ST9. In concrete, *L. monocytogenes* ST2 (serogroup IVb) has consistently been reported as one of the most common STs in studies including both clinical and food-related isolates (Mejía et al., 2023). Additionally, this ST has also been implicated in outbreaks in Europe—for example, in Germany (Halbedel et al., 2019) and is regarded as one of the sequence types most strongly associated with clinical listeriosis cases (Zhang et al., 2024). *L. monocytogenes* ST7 (serogroup IIa) was implicated in a severe Italian outbreak between May 2015 and March 2016 (Chiaverini et al., 2021). This sequence type has also been reported as the most prevalent among clinical listeriosis isolates in Norway and is frequently detected in Norwegian food industry, as well as in neighboring Nordic countries such as Sweden and Finland (Møretrø et al., 2024). In our Seeliger collection subset, however, only a single *L. monocytogenes* ST7 isolate was originated from a Nordic country, specifically from Copenhagen, Denmark, and it was recovered from a bovine placenta, rather than from a food-related source. *L. monocytogenes* ST9 (serogroup IIc) is recognized as one of the most prevalent sequence types in food and food-processing environment worldwide (Daza Prieto et al., 2024; Melero et al., 2019; Anwar et al., 2022; Li et al., 2022; Li et al., 2018). However, despite its widespread occurrence, ST9 is rarely associated with documented listeriosis outbreaks. In our Seeliger collection subset, none of the ST9 isolates originated from food sources; instead, two were of animal origin, one was from a human case, and one was of unknown source. This distribution contrasts with expectations based on the available literature and highlights the distinctive and historically rich character of the Seeliger collection.

Notably, Cluster 1 involving three different STs (ST101, ST103, ST33), was particularly interesting. Only one gene from the MLST scheme showed variation between them—specifically *Idh*. This gene corresponded to allele number 14 in ST101, 47 in ST103 and 138 in ST337, while the rest of the loci remained identical. This explains the very small allelic distance observed within Cluster 1 (3–7 allelic differences). We aligned the *Idh* gene and, interestingly, found that only a single SNP difference separated them. In the ST103 isolate, there was a G → C transversion at nucleotide position 377, whereas in the ST337 isolate, we detected a T → C transition at nucleotide position 434. All *L. monocytogenes* isolates in Cluster 1 originated from human cases reported in 1954—primarily from Germany, with only a single isolate from Canada—which likely accounts for their close genetic relatedness. In the scientific literature, *L. monocytogenes* ST101 is described as a commonly detected sequence type across various sources (Liu et al., 2009; du Toit et al., 2025). In contrast, *L. monocytogenes* ST103 and ST337 do not appear in the available literature and there are no reports linking them to the outbreaks, clinical cases or relevant food or environmental isolates.

### 4.2 Pathogenicity islands and virulence genes of *L. monocytogenes* isolates

The pathogenicity islands, particularly LIPI-1 and LIPI-3, contain key genes involved in the pathogenesis of *L. monocytogenes*, but the bacterium’s virulence is multifactorial and depends on numerous factors located outside these islands. The *L. monocytogenes* strains carrying LIPI-3 belonged to serogroup IVb, except for ST39 and ST117, which belonged to serogroup IIb. This indicates that most of the LIPI-3 positive strains were part of serogroup IVb, one of the groups most strongly associated with hypervirulence (Guidi et al., 2021). Similarly, *L. monocytogenes* strains carrying LIPI-4 also belonged to serogroup IVb, except for ST524 strains which belonged to serogroup L. Two *L. monocytogenes* ST4 strains were carrier of LIPI-3 and LIPI-4: one of the isolates was obtained from a newborn in 1954, belonging to the Seeliger collection, and the other isolate was obtained from compost in 2018, from AGES internal database. Both *L. monocytogenes* isolates formed a cluster differing by only nine alleles, despite their different temporal and ecological niches. We performed the alignment of these nine alleles and the following point mutations were revealed: one A-G transition, two T-G transversions and six T-C transitions. These mutations explain for the differences observed between the two isolates, however, we cannot establish a solid hypothesis to explain how two isolates that are so closely related genetically were recovered from such different ecological niches. These findings highlight the remarkable resilience and ecological versatility of *L. monocytogenes*.

Several studies have reported that the VGs *vip*, *inlF* and *inlK* occur exclusively in *L. monocytogenes* lineage II (Daza Prieto et al., 2024; Pyz-Lukasik et al., 2022), which is consistent with our findings on the Seeliger isolates included in our study. Among these isolates, only serogroup II—and a single *L. monocytogenes* serogroup L strain—carried *vip* and *inlK*, while *inlF* was detected exclusively in serogroup II. On the other hand, the study carried out by Bubert et al. (1997) highlighted that the product of the VG *iap*/*cwha*, the p60 protein, is essential for the viability and proper cell division of *L. monocytogenes*. Likewise, a previous study has shown that *lplA1* is required for efficient intracellular growth and cytosolic replication of the bacterium (Keeney et al., 2007). Based on this evidence, the combined absence of *iap/cwhA* and *lplA1* is expected to generate an attenuated phenotype characterized by low pathogenic potential, abnormal cell morphology (chaining). In our Seeliger isolates, none exhibited the simultaneous absence of these two virulence genes, allowing us to hypothesize that they did not show a reduced-virulence phenotype. In our previous study, Daza Prieto et al. (2024), only one out of 160 analyzed *L. monocytogenes* isolates carried the combined absence of both genes; this strain was isolated from smoked pork sausage and belonged to clonal complex CC101 (ST101). This underscores that the combined absence of both virulence genes (*iap/cwhA* and *lplA1*) is a rare occurrence. On the other hand, all analyzed Seeliger *L. monocytogenes* isolates carried the virulence regulator *prfA*, which is the central virulence gene in *L. monocytogenes*. These results contrast slightly with our previous study, in which only one *L. monocytogenes* strain (ST101) out of 160 lacked the *prfA* gene (Daza Prieto et al., 2024). Additionally, the 21 Seeliger *L. monocytogenes* serogroup IVb isolates analyzed in our study lacked the following VGs: *ami*, *aut*, *gtcA*, *inlF*, *inlJ*, *inlK* and *vip*. This pattern may indicate a slightly attenuated virulence profile—potentially reflecting reduced adhesion capacity—although this does not imply a substantially reduced virulence, as these isolates retain the essential core virulence determinants. The stress-response-related gene *sigB* was detected in all 64 *L. monocytogenes* isolates. Given that *sigB* acts a global stress regulator and plays a critical role in bacterial survival and resilience under adverse conditions (Ahmed et al., 2019), its distribution across such a broad time period suggests that these isolates are highly adapted and exhibit remarkable resilience.

Taken together, the conservation of the key pathogenicity islands and the central regulatory genes, in addition to the stress-response-related gene *sigB* support the hypothesis of a moderate-high virulence in *L. monocytogenes* as well as reflecting high stability of essential virulence determinants, which allows the bacterium to establish infection.

### 4.3 Antimicrobial resistance genes, mobile genetic elements and plasmids

The *lin* and *fosX* resistance genes were present in all analyzed Seeliger *L. monocytogenes* isolates. Both *lin* and *fosX* are intrinsic to *L. monocytogenes* and contribute to resistance against lincosamides and fosfomycin (Olaimat, 2018; Hanes and Huang, 2010; Fredriksson-Ahoma et al., 2022).

Thirty-five out of 64 studied isolates carried MGEs. Previous reports identified *ISLmo3*, *ISLmo5*, *ISLmo7*, *ISLmo9*, *ISLmo8* and *ISS1N* as some of the most common MGEs in *L. monocytogenes* (Daza Prieto et al., 2024; Parra-Flores et al., 2022). Our results are consistent with these findings, as several of these elements were also frequently detected in our study: *ISLmo7* (5/35 isolates), *ISLmo9* (10/35 isolates), *ISLmo8* (7/35 isolates) and *ISS1N* (6/35 isolates). However, the most prevalent MGEs in our dataset were *ISLmo1* (31/35 isolates), *Tn5422* (11/35 isolates) and *ISLmo4* (9/35 isolates). The MGE *Tn5422* is a natural transposon of *L. monocytogenes* that can mediate genomic deletions, which is likely one of the causes of the size variability observed among *L. monocytogenes* plasmids (Lebrun et al., 1994). *ISLmo1* is an insertion sequence in *L. monocytogenes*, and it is reported to be one of the most prevalent IS elements in this species, which aligns with our findings (Mangele et al., 2024).

In our study, the Seeliger isolates carrying plasmids belonged to ST7, ST9, ST39, ST59, ST101 and ST3397 collected between 1954–1986. Among these, *L. monocytogenes* ST9 (Mangele et al., 2024) have previously been reported in the literature as plasmid-carriers, which is consistent with our findings. For the remaining STs, no published evidence currently indicates that they harbor plasmids.

### 4.4 Presence of orthogroups in *L. monocytogenes* strains

The analysis of orthologous genes is based on identifying functionally conserved proteins, enabling researchers to predict gene functions. In addition, it plays a crucial role in evaluating virulence and antibiotic resistance by offering insights into genomic evolution and adaptation (Prieto et al., 2025). The accessory genome in this historical collection is strongly structured by phylogeny. Within-lineage correlations show that accessory-gene repertoires retain clear phylogenetic signal: isolates that are more closely related in their core genomes share more accessory content. The persistence of strong correlations within both lineages indicates that the global association is not driven solely by the deep split between Lineages I and II, although that division contributes substantially. This structure is consistent with vertical inheritance and lineage-associated gene retention, but could also arise from preferential exchange among close relatives, differential loss from a shared ancestral pool, or lineage-specific ecological selection.

Between lineages, the association disappears: on average, ~46% of accessory orthogroups in the pairwise union were not shared, essentially independently of core-genome distance. This value lies well below the Jaccard bound, so the constancy is not a metric artifact. The relationship between accessory distance and core divergence should therefore be interpreted within lineages only. We describe this band empirically; demonstrating a true plateau, rather than an absence of association over a narrow unsampled interval, would require an explicit non-linear or breakpoint model.

These findings are consistent with previous work. Lineage-specific gene content in *L. monocytogenes* has been documented since the identification of chromosomal segments present in Lineage II but absent from all Lineage I strains (Chaomei et al., 2003), and pangenome analyses have attributed most gene-scale variation to a small number of hypervariable integration hotspots, prophages and mobile elements (Kuenne et al., 2013). Our data quantify that structure, and the core genome recovered here, 2,129 orthogroups, falls within the numerical range of published core-gene estimates for the species, approximately 2,000 to 2,200 (Kuenne et al., 2013).

Orthogroups associated with plasmids, prophages and transposases occurred almost exclusively in the least widely shared part of the accessory genome, whereas transport, metabolic and cell-surface functions predominated among orthogroups carried by most isolates. This is the distribution expected if mobile elements are acquired and lost repeatedly within lineages, and therefore rarely reach high prevalence, while functions maintained by stable selection persist and become widely shared. The pattern also offers a mechanistic reading of the phylogenetic congruence reported above: gene flux concentrated in a mobile, low-prevalence fraction can proceed without disrupting the accessory genome as a whole. This interpretation is constrained by annotation rather than by the analysis. Because 77.8% of accessory orthogroups were annotated as hypothetical proteins or could not be assigned to a category, these statements rest on 251 orthogroups, and domain-based assignment would be required to establish whether the unclassified fraction follows the same distribution.

Because these isolates were collected between 1918 and 1986 (Haase et al., 2011), the year being unrecorded for three of them, and because the four most prevalent ST in the collection—ST2, ST7, ST9 and ST101—were recovered between 1949 and 1986, the results indicate that this lineage-structured organization was already established, in lineages that remain common today, well before the period of intensive food-chain surveillance from which most contemporary collections derive.

Two limitations apply. Gene presence and absence were inferred from assemblies of mixed quality, and although restricting the lineage-stratified analysis to the 47 lineage-assigned isolates with complete-genome assemblies did not alter the conclusions, absences in fragmented genomes should still be confirmed by direct sequence search before being interpreted as gene loss. Second, the analysis establishes correlation, not mechanism. In particular, isolates indistinguishable on the core genome may differ in up to 35% of the accessory orthogroups in their pairwise union, so shared accessory content alone cannot be taken as evidence of epidemiological linkage or direct transmission, especially where sampling location, date and source are incomplete.

## 5 Conclusion

Whole-genome sequencing revealed considerable genetic diversity among the oldest *L. monocytogenes* isolates of the Seeliger collection analyzed in this study. All strains carried virulence genes, including *prfA*, the master virulence regulator, together with intrinsic resistance genes, and LIPI-3, LIPI-4, plasmids and other mobile genetic elements were identified in a subset of strains. Orthogroup analysis further showed that several strains harbored highly isolate-specific orthogroups, suggesting protective mechanisms potentially associated with bacterial defense systems and with lineage-associated gene retention. Their small allelic distances from contemporary isolates underline a pronounced capacity to adapt to diverse sources and to persist over extended periods. Taken together, these historical isolates demonstrate the long-term stability of clonal lineages and the conservation of key virulence determinants, with limited accessory-genome divergence within lineages but pronounced divergence between them. These findings reinforce the need for strengthened prevention and hygiene strategies, and for deeper investigation of the accessory-genome-mediated mechanisms that enable this pathogen to colonize extreme substrates and ecological niches.

## Data Availability

The datasets presented in this study can be found in online repositories. The names of the repository/repositories and accession number(s) can be found in the article/Supplementary material.

## Footnotes

1
https://github.com/rrwick/Filtlong

2
https://www.mgc.ac.cn/cgi-bin/VFs/genus.cgi?Genus=Listeria

3
https://bigsdb.pasteur.fr/cgi-bin/bigsdb/bigsdb.pl?db=pubmlst_listeria_isolates&page=profiles

## Supplementary material

SUPPLEMENTARY TABLE 1 Information on serogroup, clonal complex (CC), sequence type (ST), country of origin, isolation source, year of isolation, mobile genetic element (MGEs) number and names, plasmid size, presence (blue)/absence (white) of Listeria pathogenicity Island (LIPI) 3 and 4 and presence (red)/absence (white) of all virulence genes (VGs) and *sigB* from 64 Seeliger *L. monocytogenes* isolates analyzed in our study, in addition to 17 Seeliger and other *L. monocytogenes* isolates available in public and from internal database (Robert Koch Institute, Germany; AGES, Austria).

SUPPLEMENTARY TABLE 2 Information on accession numbers for Sequence Read Archive (SRA), biosample and genome assembly accession numbers (WGS).

## References

[ref1] Ahmed G. Veronica G. O. Martin W. Boor K. J. (2019). Cross talk between SigB and PrfA in *Listeria monocytogenes* facilitates transitions between extra- and intracellular environments. Microbiol. Mol. Biol. Rev. 83:e00034-19. doi: 10.1128/mmbr.00034-19 PMC6759667 31484692

[ref2] Anwar T. M. Pan H. Chai W. Ed-Dra A. Fang W. Li Y. . (2022). Genetic diversity, virulence factors, and antimicrobial resistance of *Listeria monocytogenes* from food, livestock, and clinical samples between 2002 and 2019 in China. Int. J. Food Microbiol. 366:109572. doi: 10.1016/j.ijfoodmicro.2022.109572, 35176609

[ref3] Bankevich A. Nurk S. Antipov D. Gurevich A. A. Dvorkin M. Kulikov A. S. . (2012). SPAdes: a new genome assembly algorithm and its applications to single-cell sequencing. J. Comput. Biol. 19, 455–477. doi: 10.1089/cmb.2012.0021, 22506599 PMC3342519

[ref4] Benjamini Y. Hochberg Y. (1995). Controlling the false discovery rate: a practical and powerful approach to multiple testing. J. Royal Statist. Soc. Ser. B 57, 289–300. doi: 10.1111/j.2517-6161.1995.tb02031.x

[ref5] Bubert A. Kestler H. Götz M. Böckmann R. Goebel W. (1997). The *Listeria monocytogenes*iap gene as an indicator gene for the study of PrfA-dependent regulation. Mol. Gen. Genet. 256, 54–62. doi: 10.1007/s004380050545, 9341679

[ref6] Buchfink B. Reuter K. Drost H. G. (2021). Sensitive protein alignments at tree-of-life scale using DIAMOND. Nat. Methods 18, 366–368. doi: 10.1038/s41592-021-01101-x, 33828273 PMC8026399

[ref7] Chaomei Z. Min Z. Jingliang J. Joseph N. John W. Terry P. M. . (2003). Genome diversification in phylogenetic lineages I and II of *Listeria monocytogenes*: identification of segments unique to lineage II populations. J. Bacteriol. 185, 5573–5584. doi: 10.1128/jb.185.18.5573-5584.2003 PMC193770 12949110

[ref8] Chiaverini A. Guidi F. Torresi M. Acciari V. A. Centorotola G. Cornacchia A. . (2021). Phylogenetic analysis and genome-wide association study applied to an Italian *Listeria monocytogenes* outbreak. Front. Microbiol. 12:750065. doi: 10.3389/fmicb.2021.750065 PMC8600327 34803971

[ref9] Daza Prieto B. Pietzka A. Martinovic A. Ruppitsch W. Zuber Bogdanovic I. (2024). Surveillance and genetic characterization of *Listeria monocytogenes* in the food chain in Montenegro during the period 2014–2022. Front. Microbiol. 15:1418333. doi: 10.3389/fmicb.2024.1418333 PMC11324475 39149205

[ref10] Doumith M. Jacquet C. Gerner-Smidt P. Graves L. M. Loncarevic S. Mathisen T. . (2005). Multicenter validation of a multiplex PCR assay for differentiating the major *Listeria monocytogenes* Serovars 1/2a, 1/2b, 1/2c, and 4b: toward an international standard. J. Food Prot. 68, 2648–2650. doi: 10.4315/0362-028X-68.12.2648, 16355837

[ref11] du Toit S. A. Gouws P. A. Rip D. (2025). Profiling *Listeria monocytogenes* in hummus, fresh produce, and food processing environments in the Western Cape, South Africa. Microbiology 14:e70060. doi: 10.1002/mbo3.70060, 40922468 PMC12417568

[ref12] EFSA (2023). Prolonged multi-country cluster of *Listeria monocytogenes* ST155 infections linked to ready-to-eat fish products. Available online at: https://www.ecdc.europa.eu/sites/default/files/documents/listeria-monocytogenes-ST155-infections-fish-products_0.pdf (Accessed May 5, 2026).

[ref13] Emms D. M. Kelly S. (2019). OrthoFinder: phylogenetic orthology inference for comparative genomics. Genome Biol. 20:238. doi: 10.1186/s13059-019-1832-y, 31727128 PMC6857279

[ref14] Enright A. J. Van Dongen S. Ouzounis C. A. (2002). An efficient algorithm for large-scale detection of protein families. Nucleic Acids Res. 30, 1575–1584. doi: 10.1093/nar/30.7.1575, 11917018 PMC101833

[ref15] Esposito C. Cardillo L. Borriello G. Ascione G. Valvini O. Galiero G. . (2021). First detection of *Listeria monocytogenes* in a buffalo aborted foetus in Campania region (southern Italy). Front. Vet. Sci. 7:571654. doi: 10.3389/fvets.2020.571654 PMC7902923 33644140

[ref16] Fredriksson-Ahoma M. Sauvala M. Kurittu P. Heljanko V. Heikinheimo A. Paulsen P. (2022). Characterisation of *Listeria monocytogenes* isolates from hunted game and game meat from Finland. Foods. 11:3679. doi: 10.3390/foods11223679 PMC9689155 36429271

[ref17] Guidi F. Chiaverini A. Repetto A. Lorenzetti C. Centorotola G. Bazzucchi V. . (2021). Hyper-virulent *Listeria monocytogenes* strains associated with respiratory infections in central Italy. Front. Cell. Infect. Microbiol. 11:765540. doi: 10.3389/fcimb.2021.765540 PMC8564288 34746033

[ref18] Haase J. K. Murphy R. A. Choudhury K. R. Achtman M. (2011). Revival of Seeliger’s historical “special *Listeria* culture collection”. Environ. Microbiol. 13, 3163–3171. doi: 10.1111/j.1462-2920.2011.02610.x 22003999

[ref19] Halbedel S. Fischer M. A. Thürmer A. Flieger A. (2021). Closed genome sequences of clinical *Listeria monocytogenes* PCR serogroup IVb isolates associated with two recent large listeriosis outbreaks in Germany. Microbiol. Resour. Announc. 10:e01434-20. doi: 10.1128/mra.01434-20 PMC7862961 33541883

[ref20] Halbedel S. Prager R. Banerji S. Kleta S. Trost E. Nishanth G. . (2019). A *Listeria monocytogenes* ST2 clone lacking chitinase ChiB from an outbreak of non-invasive gastroenteritis. Emerg Microbes Infect. 8, 17–28. doi: 10.1080/22221751.2018.1558960, 30866756 PMC6455121

[ref21] Hanes R. M. Huang Z. (2010). Investigation of antimicrobial resistance genes in *Listeria monocytogenes* from 2010 through to 2021. Int. J. Environ. Res. Public Health 19:5506. doi: 10.3390/ijerph19095506, 35564901 PMC9099560

[ref22] Hernandez-Milian A. Payeras-Cifre A. (2014). What is new in listeriosis? Biomed. Res. Int. 2014:358051. doi: 10.1155/2014/358051, 24822197 PMC4005144

[ref23] Hope A. C. A. (1968). A simplified Monte Carlo significance test procedure. J. Royal Statist. Soc. Ser. B 30, 582–598. doi: 10.1111/j.2517-6161.1968.tb00759.x

[ref24] Johansson M. H. K. Bortolaia V. Tansirichaiya S. Aarestrup F. M. Roberts A. P. Petersen T. N. (2021). Detection of mobile genetic elements associated with antibiotic resistance in *Salmonella enterica* using a newly developed web tool: MobileElementFinder. J. Antimicrob. Chemother. 76, 101–109. doi: 10.1093/jac/dkaa390, 33009809 PMC7729385

[ref25] Keeney K. M. Stuckey J. A. O’Riordan M. X. D. (2007). LplA1-dependent utilization of host lipoyl peptides enables Listeria cytosolic growth and virulence. Mol. Microbiol. 66, 758–770. doi: 10.1111/j.1365-2958.2007.05956.x, 17908209 PMC2367003

[ref26] Kolmogorov M. Yuan J. Lin Y. Pevzner P. A. (2019). Assembly of long, error-prone reads using repeat graphs. Nat. Biotechnol. 37, 540–546. doi: 10.1038/s41587-019-0072-8, 30936562

[ref27] Kruskal W. H. Wallis W. A. (1952). Use of ranks in one-criterion variance analysis. J. Am. Stat. Assoc. 47, 583–621. doi: 10.1080/01621459.1952.10483441

[ref28] Kuenne C. Billion A. Mraheil M. A. Strittmatter A. Daniel R. Goesmann A. . (2013). Reassessment of the *Listeria monocytogenes*pan-genome reveals dynamic integration hotspots and mobile genetic elements as major components of the accessory genome. BMC Genomics 14:47. doi: 10.1186/1471-2164-14-47, 23339658 PMC3556495

[ref29] Lebrun M. Audurier A. Cossart P. (1994). Plasmid-borne cadmium resistance genes in *Listeria monocytogenes* are present on Tn5422, a novel transposon closely related to Tn917. J. Bacteriol. 176, 3049–3061. doi: 10.1128/jb.176.10.3049-3061.1994, 8188606 PMC205463

[ref30] Lee S. Ward T. J. Graves L. M. Wolf L. A. Sperry K. Siletzky R. M. . (2012). Atypical *Listeria monocytogenes* serotype 4b strains harboring a lineage II-specific gene cassette. Appl. Environ. Microbiol. 78, 660–667. doi: 10.1128/aem.06378-11, 22138999 PMC3264116

[ref31] Lefort V. Desper R. Gascuel O. (2015). FastME 2.0: a comprehensive, accurate, and fast distance-based phylogeny inference program. Mol. Biol. Evol. 32, 2798–2800. doi: 10.1093/molbev/msv150 PMC4576710 26130081

[ref32] Li W. Bai L. Fu P. Han H. Liu J. Guo Y. (2018). The epidemiology of *Listeria monocytogenes* in China. Foodborne Pathog. Dis. 15, 459–466. doi: 10.1089/fpd.2017.2409, 30124341

[ref33] Li W. O’Neill K. R. Haft D. H. DiCuccio M. Chetvernin V. Badretdin A. . (2021). RefSeq: expanding the prokaryotic genome annotation pipeline reach with protein family model curation. Nucleic Acids Res. 49, D1020–D1028. doi: 10.1093/nar/gkaa1105, 33270901 PMC7779008

[ref34] Li X. Shi X. Song Y. Yao S. Li K. Shi B. . (2022). Genetic diversity, antibiotic resistance, and virulence profiles of *Listeria monocytogenes* from retail meat and meat processing. Food Res. Int. 162:112040. doi: 10.1016/j.foodres.2022.112040, 36461311

[ref35] Liu T.-P. Lin L.-C. Chang S.-C. Ou Y.-H. Lu J.-J. (2009). Molecular characteristics and virulence profile of clinical *Listeria monocytogenes* isolates in northern Taiwan, 2009–2019. Foodborne Pathog. Dis. 21, 386–394. doi: 10.1089/fpd.2023.0136, 38346310

[ref36] Liu B. Zheng D. Zhou S. Chen L. Yang J. (2022). VFDB 2022: a general classification scheme for bacterial virulence factors. Nucleic Acids Res. 50:D912. doi: 10.1093/nar/gkab1107, 34850947 PMC8728188

[ref37] Magalhaes R. Almeida G. Ferreira V. Santos I. Silva J. Mendes M. M. . (2015). Cheese-related listeriosis outbreak, Portugal, march 2009 to February 2012. Surveill. Outbreak Rep. 20:21104. doi: 10.2807/1560-7917.es2015.20.17.21104 25955775

[ref38] Mangele A. Adesiyun A. Mafuna T. Pierneef R. Moerane R. Gcebel N. (2024). Virulence potential and antimicrobial resistance of *Listeria monocytogenes* isolates obtained from beef and beef-based products deciphered using whole-genome sequencing. Microorganisms. 12:1166. doi: 10.3390/microorganisms12061166 PMC11205329 38930548

[ref39] Mantel N. (1967). The detection of disease clustering and a generalized regression approach. Cancer Res. 27, 209–220. Available at: https://aacrjournals.org/cancerres/article/27/2_Part_1/209/476508/The-Detection-of-Disease-Clustering-and-a 6018555

[ref40] Maung A. T. Abdelaziz M. N. S. Mohammadi T. N. Zhao J. EI-Telbany M. Nakayama M. . (2023). Comparison of prevalence, characterization, antimicrobial resistance and pathogenicity of foodborne *Listeria monocytogenes* in recent 5 years in Japan. Microb. Pathog. 183:106333. doi: 10.1016/j.micpath.2023.106333, 37673352

[ref41] Mejía L. Espinosa-Mata E. Freire A. L. Zapata S. González-Candelas F. (2023). *Listeria monocytogenes*, a silent foodborne pathogen in Ecuador. Front. Microbiol. 14:1278860. doi: 10.3389/fmicb.2023.1278860 PMC10764610 38179446

[ref42] Melero B. Manso B. Stessl B. Hernández M. Wagner M. Rovira J. . (2019). Distribution and persistence of *Listeria monocytogenes* in a heavily contaminated poultry processing facility. J. Food Prot. 82, 1524–1531. doi: 10.4315/0362-028X.JFP-19-087, 31414898

[ref43] Michael F. Vyacheslav B. Haft D. H. Prasad A. B. Slotta D. J. Tolstoy I. . (2019). Validating the AMRFinder tool and resistance gene database by using antimicrobial resistance genotype-phenotype correlations in a collection of isolates. Antimicrob. Agents Chemother. 63:e00483-19. doi: 10.1128/aac.00483-19 PMC6811410 31427293

[ref44] Michel D. Carmen B. Philippe G. Christine J. Paul M. (2004). Differentiation of the major *Listeria monocytogenes* serovars by multiplex PCR. J. Clin. Microbiol. 42, 3819–3822. doi: 10.1128/jcm.42.8.3819-3822.2004 PMC497638 15297538

[ref45] Møretrø T. Wagner E. Heir E. Langsrud S. Fagerlund A. (2024). Genomic analysis of *Listeria monocytogenes* CC7 associated with clinical infections and persistence in the food industry. Int. J. Food Microbiol. 410:110482. doi: 10.1016/j.ijfoodmicro.2023.110482, 37977076

[ref46] Moura A. Criscuolo A. Pouseele H. Maury M. M. Leclercq A. Tarr C. . (2016). Whole genome-based population biology and epidemiological surveillance of *Listeria monocytogenes*. Nat. Microbiol. 2:16185. doi: 10.1038/nmicrobiol.2016.185, 27723724 PMC8903085

[ref47] Olaimat A. (2018). Emergence of antibiotic resistance in *Listeria monocytogenes* isolated from food products: a comprehensive review. Compr. Rev. Food Sci. Food Saf. 17, 1277–1292. doi: 10.1111/1541-4337.12387, 33350166

[ref48] Paradis E. Schliep K. (2019). Ape 5.0: an environment for modern phylogenetics and evolutionary analyses in R. Bioinformatics 35, 526–528. doi: 10.1093/bioinformatics/bty633 30016406

[ref49] Parra-Flores J. Holý O. Bustamante F. Lepuschitz S. Pietzka A. Contreras-Fernández A. . (2022). Virulence and antibiotic resistance genes in *Listeria monocytogenes* strains isolated from ready-to-eat foods in Chile. Front. Microbiol. 12:796040. doi: 10.3389/fmicb.2021.796040 PMC8921925 35299835

[ref50] Poyart-Salmeron C. Carlier C. Trieu-Cuot P. Courvalin P. Courtieu A. L. (1990). Transferable plasmid-mediated antibiotic resistance in *Listeria monocytogenes*. Lancet 335, 1422–1426. doi: 10.1016/0140-6736(90)91447-I, 1972210

[ref51] Prieto B. D. Raicevic N. Hyden P. Cabal A. Ladstätter J. Richter S. . (2025). *Ewingella docleensis* sp. nov. isolated from artisanal Montenegrin komanski cheese. The Microbe 8:100449. doi: 10.1016/j.microb.2025.100449

[ref52] Pyz-Lukasik R. Paszkiewicz W. Kielbus M. Ziomek M. Gondek M. Domaradzki P. . (2022). Genetic diversity and potential virulence of *Listeria monocytogenes* isolates originating from polish artisanal cheeses. Foods. 11:2805. doi: 10.3390/foods11182805, 36140933 PMC9497517

[ref53] Ragon M. Wirth T. Hollandt F. Lavenir R. Lecuit M. Le Monnier A. . (2008). A new perspective on *Listeria monocytogenes* evolution. PLoS Pathog. 4:e1000146. doi: 10.1371/journal.ppat.1000146, 18773117 PMC2518857

[ref54] Robertson J. Nash J. H. E. (2018). MOB-suite: software tools for clustering, reconstruction and typing of plasmids from draft assemblies. Microb Genom. 4:e000206. doi: 10.1099/mgen.0.000206, 30052170 PMC6159552

[ref55] Salcedo C. Arreaza L. Alcalá B. de la Fuente L. Vázquez J. A. (2003). Development of a multilocus sequence typing method for analysis of *Listeria monocytogenes* clones. J. Clin. Microbiol. 41, 757–762. doi: 10.1128/jcm.41.2.757-762.2003 PMC149676 12574278

[ref56] Tayeb Bizhar A. Mohamed-Sharif Y. H. Choli F. R. Haji S. S. Ibrahim M. M. Haji S. K. . (2023). Antimicrobial susceptibility profile of *Listeria monocytogenes* isolated from meat products: a systematic review and meta-analysis. Foodborne Pathog. Dis. 20, 315–333. doi: 10.1089/fpd.2023.0004 37389828

[ref57] Werner R. Ariane P. Karola P. Stefan B. Lasa F. H. Franz A. . (2015). Defining and evaluating a core genome multilocus sequence typing scheme for whole-genome sequence-based typing of *Listeria monocytogenes*. J. Clin. Microbiol. 53, 2869–2876. doi: 10.1128/jcm.01193-15 PMC4540939 26135865

[ref58] Zhang P. Ji L. Wu X. Chen L. Yan W. Dong F. (2024). Prevalence, genotypic characteristics, and antibiotic resistance of *Listeria monocytogenes* from retail foods in Huzhou, China. J. Food Prot. 87:100307. doi: 10.1016/j.jfp.2024.100307, 38797247

